# *EjTFL1* Genes Promote Growth but Inhibit Flower Bud Differentiation in Loquat

**DOI:** 10.3389/fpls.2020.00576

**Published:** 2020-05-15

**Authors:** Yuanyuan Jiang, Yunmei Zhu, Ling Zhang, Wenbing Su, Jiangrong Peng, Xianghui Yang, Huwei Song, Yongshun Gao, Shunquan Lin

**Affiliations:** ^1^Henry Fok College of Biology and Agriculture, Shaoguan University, Shaoguan, China; ^2^Key Laboratory of South China Horticultural Crop Biology and Germplasm Enhancement, Ministry of Agriculture, College of Horticulture, South China Agricultural University, Guangzhou, China; ^3^Jiangsu Key Laboratory for Eco-Agricultural Biotechnology Around Hongze Lake, Huaiyin Normal University, Huai’an, China; ^4^Beijing Academy of Forestry and Pomology Sciences, Beijing Academy of Agriculture and Forestry Sciences, Beijing, China

**Keywords:** loquat, *EjTFL1*, flowering time, GA_3_, *TERMINAL FLOWER1*

## Abstract

*TERMINAL FLOWER1* (*TFL1*), a key factor belonging to the phosphatidyl ethanolamine-binding protein (PEBP) family, controls flowering time and inflorescence architecture in some plants. However, the role of *TFL1* in loquat remains unknown. In this study, we cloned two *TFL1*-like genes (*EjTFL1-1* and *EjTFL1-2*) with conserved deduced amino acid sequences from cultivated loquat (*Eriobotrya japonica* Lindl.). First, we determined that flower bud differentiation occurs at the end of June and early July, and then comprehensively analyzed the temporal and spatial expression patterns of these *EjTFL1s* during loquat growth and development. We observed the contrasting expression trends for *EjTFL1s* and *EjAP1s (APETALA 1)* in shoot apices, and *EjTFL1s* were mainly expressed in young tissues. In addition, short-day and exogenous GA_3_ treatments promoted the expression of *EjTFL1s*, and no flower bud differentiation was observed after these treatments in loquat. Moreover, EjTFL1s were localized to the cytoplasm and nucleus, and both interacted with another flowering transcription factor, EjFD, in the nucleus, and EjTFL1s-EjFD complex significantly repressed the promoter activity of *EjAP1-1*. The two *EjTFL1s* were overexpressed in wild-type *Arabidopsis thaliana* Col-0, which delayed flowering time, promoted stem elongation, increased the number of branches, and also affected flower and silique phenotypes. In conclusion, our results suggested that *EjTFL1-1* and *EjTFL1-2* do not show the same pattern of expression whereas both are able of inhibiting flower bud differentiation and promoting vegetative growth in loquat by integrating GA_3_ and photoperiod signals. These findings provide useful clues for analyzing the flowering regulatory network of loquat and provide meaningful references for flowering regulation research of other woody fruit trees.

## Introduction

Flowering in angiosperm plants marks the change from the vegetative to the reproductive phase, and the time of floral initiation is regulated by environmental (photoperiod and temperature) and endogenous (age, autonomous and GA) pathways (Wellmer and Riechmann, 2010; Andres and Coupland, 2012). In *Arabidopsis*, these signaling pathways converge on the floral integrator gene *FLOWERING LOCUS T* (*FT*), which belongs to PEBP gene family that also includes *TERMINAL FLOWER 1* (*TFL1*), *MOTHER OF FT AND TFL1-like* (*MFT*), *BROTHER OF FT(BFT)*, *TWIN SISTER OF FT* (*TSF*), and *Arabidopsis thaliana RELATIVE OF CENTRORADIALIS* (*ATC*) (Karlgren et al., 2011; Wickland and Hanzawa, 2015). *FT* and *TSF* are floral activators (Yamaguchi et al., 2005; Jang et al., 2009) whereas *TFL1*, *BFT* and *ATC* act as floral inhibitors (Bradley et al., 1997; Yoo et al., 2010; Huang et al., 2012). FT is expressed in leaves and then transported to the shoot apices meristems to positively regulate downstream flowering genes and to induce floral differentiation and flowering (Kobayashi and Weigel, 2007; Liu et al., 2012).

Although *TFL1* and *FT* genes belong to the same family and are highly homologous, differences in key amino acids lead to dissimilar functions (Hanzawa et al., 2005; Ahn et al., 2006; Ho and Weigel, 2014). His88/Asp144 in *TFL1* and corresponding Tyr85/Gln140 in *FT* are the most critical residues for functional divergence between *TFL1* and *FT*.

In the *Arabidopsis*, both FT and TFL1 bind to FD but play different roles. Binding of FT to the transcription factor FD produces a strong activator that promotes expression of *AP1* and flowering, and the complex of FD and TFL1 serves as a strong inhibitor of downstream flower meristem identity genes such as *AP1* and *LFY*, which delay flowering (Ratcliffe et al., 1998, 1999; Abe et al., 2005; Ahn et al., 2006; Hanano and Goto, 2011). The cloning and functional analysis of the *TFL1* gene in other plants, such as apple (Kotoda and Wada, 2005; Mimida et al., 2009) pear (Freiman et al., 2012; Wang and Pijut, 2013) strawberry (Iwata et al., 2012; Koskela et al., 2012, 2016, 2017; Nakano et al., 2015), poplar (Igasaki et al., 2008; Ruonala et al., 2008; Mohamed et al., 2010) and rose (Iwata et al., 2012; Randoux et al., 2014), has been conducted. Silencing of *MdTFL1* significantly accelerates flowering in transformed apple plants (Flachowsky et al., 2012; Yamagishi et al., 2016; Charrier et al., 2019) and overexpression of the apple *TFL1* gene in *Arabidopsis* delays flowering (Kotoda and Wada, 2005). Similarly, silencing of pear *TFL1* caused early flowering (Freiman et al., 2012; Yamagishi et al., 2016; Charrier et al., 2019). These studies indicated that the function of *TFL1* is relatively conserved among different species, mainly as a flowering inhibitory factor. However, there are no reports on the functional verification of *TFL1* homologous genes in loquat.

Loquat (*Eriobotrya japonica* Lindl.), which belongs to the Maloideae subfamily of the Rosaceae family, is a tropical and subtropical evergreen fruit tree. In Rosaceae fruit trees such as apples, pears, and plums, the time of flower bud differentiation and flowering do not occur in the same year (Kurokura et al., 2013). After flower bud differentiation, a period of dormancy is required, and flowering begins in the next year (Kurokura et al., 2013). However, the flower bud differentiation and flowering time of loquat do occur in the same year (Lin, 2007) as confirmed by Jiang et al. (2019): flower bud differentiation of loquat occurred at the end of June, and the flower buds continued to develop until flowering in November-December. This unique flower development pattern is very important and interesting and may provide different perspectives for exploring the flower development pathway of Rosaceae; nonetheless, there are few reports on flower development in loquat. *EjLFY*, *EjAP1*, *EjFT*, and *EjSOC1* have been cloned from cultivated loquat (Esumi et al., 2005; Liu et al., 2013; Reig et al., 2017; Jiang et al., 2019); and *EdGI*, *EdCO*, *EdFT*, and *EdFD* have been cloned from wild loquat *Eriobotrya deflexa* Nakai forma *koshunensis* (Zhang L. et al., 2016; Zhang et al., 2019).

Although two *TFL1* homologues have been cloned from cultivated loquat (Esumi et al., 2005) their expression patterns and roles in the regulation of flowering have not yet been elucidated. In this study, two *TFL1* homologues were isolated from loquat, namely *EjTFL1-1* and *EjTFL1-2*. To explore their functions during the growth and development of loquat, we comprehensively analyzed their temporal and spatial expression patterns and tested their ability to interact with EjFD using BiFC and the yeast two-hybrid assay. In addition, we examined the effects of gibberellin and photoperiod signaling on the expression patterns of *EjTFL1* and overexpressed both *EjTFL1s* in *Arabidopsis* for functional analysis.

## Materials and Methods

### Plant Materials

Tissue samples were collected from ‘Jiefangzhong’ loquat (*Eriobotrya japonica* Lindl.) field-gown in the loquat germplasm resource preservation garden, South China Agricultural University (Guangzhou, China N23°09′N,113°20′E). The trees used in the experiment were 12-year-old trees, and showed normal flowering. The 5th-6th leaves with the same maturity at the upper end of the stem in the same period (the leaves turned into dark green as the standard), and the young leaves, buds (terminal buds), flowers and fruits were collected in the same period and the phenotype was consistent (tissues were taken at 16:00). All samples for quantitative analysis were frozen with liquid nitrogen immediately after collection and then stored at −80°C until use. Three independent experiments were conducted from three independent trees. Year-round follow-up observations, and sampling were conducted every 2 weeks, and paraffin sections of the shoot apices meristems were observed using fluorescence microscope (Observer. D1, Zeiss, Germany) in a bright-field channel and photographed color images. *Arabidopsis thaliana* wild-type Col-0 was used in this study. *Arabidopsis* and *Nicotiana benthamiana* were grown under long-day conditions (16 h light/8 h dark cycle) at 22°C.

### Total RNA Extraction and Reverse-Transcription and Genomic DNA Extraction

Total RNA from flowers, fruits, stems, leaves and buds of ‘Jiefanzhong’ was extracted using EASY Spin Plus Plant RNA Extraction Kit (Aidlab, China), and total RNA from whole *Arabidopsis* plants was extracted using HiPure Plant RNA Mini Kit (Magen, China). First-strand cDNA was synthesized using PrimeScript^TM^ RT reagent Kit with gDNA Eraser (TaKaRa, Japan). Genomic DNA was extracted from loquat buds using HiPure Plant DNA Mini Kit (Magen, China).

### Gene and Promoter Cloning and Sequence Analysis

*EjTFL1-1*, *EjTFL1-2*, and *EjFD* cDNA and the promoter regions of *EjTFL1-1*, *EjTFL1-2*, *EjAP1-1*, and *EjAP1-2* were amplified using Prime STAR^®^ Max DNA Polymerase (TaKaRa, Japan) with the primer sequences listed in Supplementary Table S1; the fragments were inserted into the pGEM-T easy vector (Promega, United States). Sequence information was obtained from the *de novo* genome sequencing project of loquat by our research group, which has not yet been published. Amino acid sequences were aligned using Clustal X and Gene Doc. A phylogenetic tree was generated using MEGA 6 by the neighbor-joining (N-J) method with 1000 bootstrap replications. Predictive analysis of cis-acting elements of the promoter fragments was performed online at PlantCARE.^1^

### Expression Analysis

iTaqTM universal SYBR Green Super mix kit (Bio-Rad, United States) was used for qRT-PCR (quantitative real time polymerase chain reaction) in the LightCyclerR 480 system (Roche, United States). qRT-PCR experiments and analyses were carried out as described by Zhang L. et al. (2016). Gene expression levels were normalized using *Ejβ-Actin* for loquat (Shan et al., 2008) and *AtTUB2*(AT5G62690) for *Arabidopsis* (Yan et al., 2014). The primer sequences used in real-time PCR are listed in Supplementary Tables S2, S3. Data were collected from three biological replicates.

### GA_3_ and Short-Day Treatments

For short-day treatments, the shading was set for 8 h (10:00–18:00) of natural light and 16 h of full darkness (18:00–10:00 [second day]) each day; no shading was performed for control plants. For GA_3_ treatments, plants were sprayed once every 2 weeks with a 300 mg L^–1^ aqueous solution of GA_3_ (DingGuo, China); 0.1% (v/v) phosphoric acid and 0.025% (v/v) Triton X-100 were added as surfactants; the control plants were sprayed with 0.1% (v/v) phosphoric acid and 0.025% (v/v) Triton X-100 aqueous solution. The two experimental treatment times were from 18th May to 10th August. Three independent experiments were conducted.

### Yeast Two-Hybrid (Y2H) Assay

We used the Matchmaker Gold Yeast two-hybrid system for Y2H assay (Clontech). AD-EjTFL1 (full-length EjTFL1-1 and EjTFL1-2 fused to pGADT7) and BD-EjFD (full-length EjFD fused to pGBKT7) plasmids were constructed for the Y2H assay. The experimental procedure was carried out in strict accordance with the manufacturer’s instructions. The primer sequences used in plasmid construction are listed in Supplementary Table S4.

### Subcellular Localization and Bimolecular Fluorescence Complementation (BiFC) Assay

*EjTFL1-1*, *EjTFL1-2*, and *EjFD* were amplified by PCR and cloned into pGreen-35S-GFP (Lee et al., 2012) for subcellular localization analysis. For the BiFC assay, PCR-amplified *EjTFL1-1, EjTFL1-2*, and *EjFD* were cloned into pGreen-35S-cYFP/nYFP vectors (Hou et al., 2014). Primer sequences used in the plasmid construction are listed in Supplementary Table S4. The 35S-YFP-C-EjFD + 35S-EjTFL1s-YFP-N, 35S-YFP-C-EjFD + 35S-YFP-N, 35S-YFP-C + 35S-EjTFL1s-YFP-N, 35S-EjTFL1s-GFP, and 35S-EjFD-GFP fusion constructs were transiently transformed into *N. benthamiana* leaves using an *Agrobacterium tumefaciens* GV3101::pSoup-mediated technique, as previously described (Sparkes et al., 2006). Two days later, fluorescent signals were observed by fluorescence microscopy (Observer. D1, Zeiss, Germany).

### Dual-Luciferase Assay

The *EjAP1-1* and *EjAP1-2* promoters were cloned into the pGreenII 0800-LUC double-reporter vector as reporters. The reporter constructs were fused to LUC luciferase and REN luciferase driven by CaMV35S; *EjTFL1s* and *EjFD* were cloned into the pGreenII62-SK vector as effectors driven by CaMV35S (Hellens et al., 2005). The primer sequences used in vector construction are listed in Supplementary Table S4. The constructed effector and reporter plasmids with different combinations were co-transformed into tobacco leaves using the *A. tumefaciens* strain GV3101::pSoup method (Hellens et al., 2005). After 48–72 h, LUC and REN luciferases were quantified using the Dual Luciferase Assay kit (Promega, United States) with a Luminoskan Ascent Microplate Luminometer (Thermo, United States). The binding activities of the effectors and reporters were calculated by the ratio of LUC/REN.

### *Arabidopsis* Transformation

*EjTFL1-1* and *EjTFL1-2* were amplified by PCR and cloned into pGreen-35S (Lee et al., 2012). The constructed vectors were transformed into *A. tumefaciens* GV3101::pSoup and introduced into *A. thaliana* wild-type Col-0 using the floral dip method (Zhang et al., 2006). Transformed plants were selected on soil with Basta.

## Results

### Identification of *EjTFL1s* From *Eriobotrya japonica* Lindl

**FIGURE 1:**
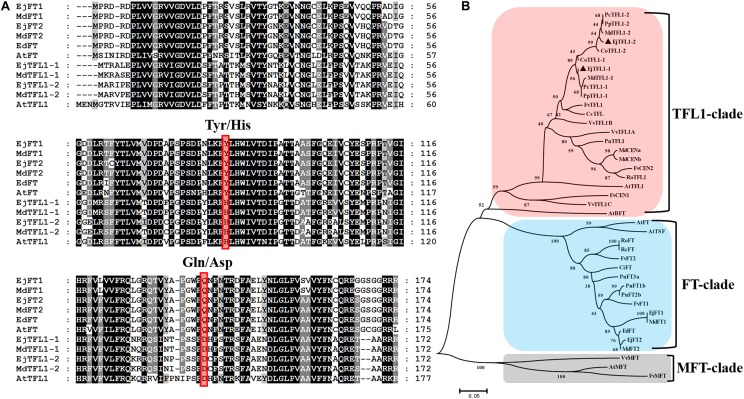
Sequence and phylogenetic analyses of EjTFL1-1 and EjTFL1-2 (At, *Arabidopsis thaliana*; Ed, *Eriobotrya deflexa*; Ej, *Eriobotrya japonica*; Md, *Malus domestica*; Fv, *Fragaria vesca*; Ro, *Rosa lucieae*; Rc, *Rosa chinensi*s; Ci, *Citrus unshiu*; Pn, *Populus nigra*; Pc, *Pyrus communis*; Cs, *Citrus sinensis*; Pp, *Pyrus pyrifolia*; Co, *Cydonia oblonga*; Vv, *Vitis vinifera*). **(A)** Amino acid sequence alignment of TFL1 and FT proteins of loquat and other species. The red box indicates the key amino acids Tyr85/His88 and Gln140/Asp144 that differ in function between TFL1 and FT. **(B)** Phylogenetic analysis of PEBP gene family proteins from loquat and other species. The protein sequences used in this study were retrieved from NCBI. Accession IDs: AtFT (BAA77838), AtTFL1 (AAB41624), AtBFT (Q9FIT4), AtTSF(BAD93590), AtMFT (Q9XFK7), EdFT (AMB72867), EjFT1(ALA56299), EjFT2 (ALA56300), MdFT1 (BAD08340), MdFT2 (BAI77728), MdTFL1-1 (BAD10961), MdTFL1-2 (BAD10967), MdCENa (BAG31957), MdCENb (BAG31958), FvFT1 (AEP23098), FvFT2 (XP_004297273), FvTFL1 (AEP23097), FvCEN1 (XP_004308107), FvCEN2 (XP_004291610), FvMFT (XP_004299541), RoFT (CAX46426), RoTFL1 (CAX46398), RcFT (CBY25182), CiFT (BAA77836), PnFT1b (BAD08338), PnFT2b (BAD08337), PnFT3a (BAD08336), PnTFL1 (BAG12897), PcTFL1-1 (BAD10963), PcTFL1-2 (BAD10969), CsTFL (AAR04683), PpTFL1-1 (BAD10962), PpTFL1-2 (BAD10968), CoTFL1-1 (BAD10964), CoTFL1-2 (BAD10970), VvTFL1A (ABI99466), VvTFL1B (ABI99467), VvTFL1C (ABI99468), VvMFT (ABI99469).

Two *TFL1* homologs, *EjTFL1-*1 and *EjTFL1-2*, were isolated from loquat buds, and the specific primers were designed using data from the unpublished loquat genome sequence. The length of *EjTFL1-1* and *EjTFL1-2* CDSs were 519 bp, encoding putative proteins of 172 amino acids that belong to the PEBP family (see Supplementary Sequences). Their sequences were highly similar, with amino acids sequence identity of 90.7%. Amino acid sequence analysis revealed that loquat EjTFL1s and EjFTs are highly similar (Figure 1A). Phylogenetic tree analysis revealed that EjTFL1s is closely related to TFL1 of the sub-family Maloideae, which clustered together into a small clade, and grouped with other plants of Rosaceae family (Figure 1B).

Alignment of the amino acid sequences showed that the EjTFL1s are similar to MdTFL1s (98% identity to MdTFL1-1 for EjTFL1-1 and 98% identity to MdTFL1-2 for EjTFL1-2). EjTFL1-1 and EjTFL1-2 contain His84 and Asp139 residues, which are conserved among other plant species and clearly distinguish *FT* homologous genes from other species, including loquat (Figure 1A). The EjTFL1 structure suggests a functional conservation. Moreover, phylogenetic tree also revealed that there is a very close genetic evolutionary relationship between loquat, apple and other Rosaceae plants.

### Analysis of *EjTFL1* Expression Patterns in Loquat

**FIGURE 2:**
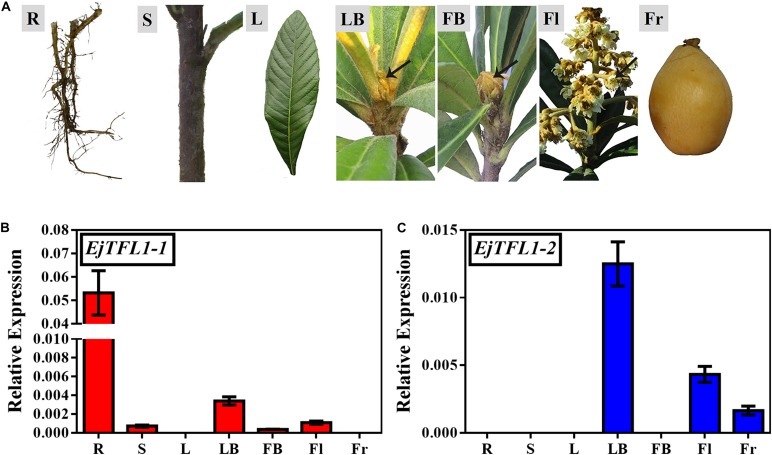
Tissue-specific expression of *EjTFL1-1* and *EjTFL1-2.* **(A)** Different tissues from loquat (*Eriobotrya japonica* Lindl) were analyzed. **(B,C)** Relative expression of *EjTFL1-1* and *EjTFL1-2* in different tissues shown in **(A)** (error bars indicate ± SE from three biological replicates). R, root (from rootstock); S, shoot; L, leaf (5.26); LB, leaf bud (5.26); FB, flower bud (8.18); Fl, flower (12.8); Fr, fruit (3.30). Leaf bud, flower bud and flower are phenotypes of the shoot apex at different developmental stages.

To explore the function and regulatory mechanisms of *EjTFL1*s, we analyzed the relative expression levels of *EjTFL1-1* and *EjTFL1-2* in different tissues of loquat (Figure 2A). *EjTFL1-1* was mainly expressed in roots and leaf buds, but the expression was relatively low in shoots, flower buds and flowers, with no expression in mature leaves and mature fruits (Figure 2B). *EjTFL1-2* was mainly expressed in leaf buds, flowers, and fruits and was not expressed in the other tissues (Figure 2C). Thus, the two *EjTFL1s* exhibit different tissue-specific expressions, which also suggests that they may have functional differences with regard to the growth and development of loquat.

### Expression Pattern of *EjTFL1s* During Growth and Development of Loquat

**FIGURE 3:**
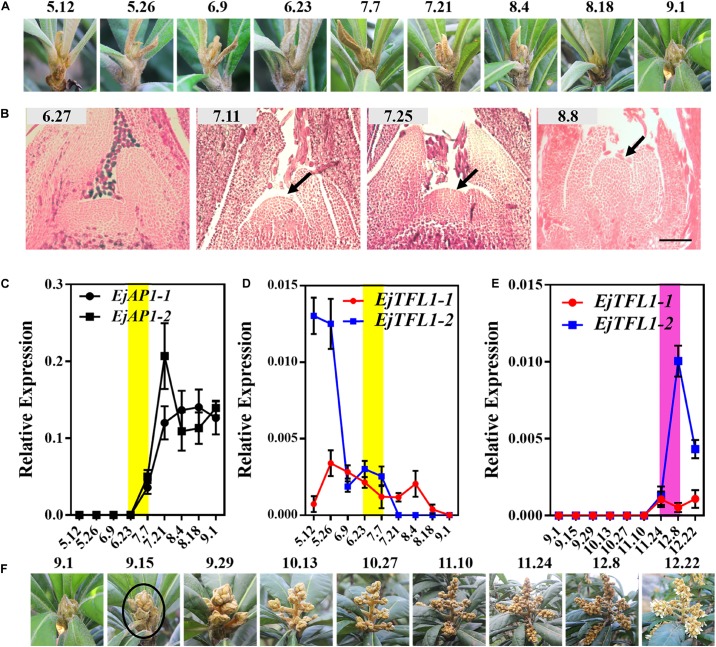
Expression patterns of *EjTFL1-1* and *EjTFL1-2* in the shoot apices. **(A)** The phenotype of the shoot apices from May to September. **(B)** Paraffin sections of the shoot apices from June to August. **(C)** Relative expression levels of the flower meristem-specific genes *EjAP1-1* and *EjAP1-2* in the shoot apices from May to September. **(D)** Relative expression levels of *EjTFL1-1* and *EjTFL1-2* in the shoot apices from May to September. **(E)** Relative expression levels of *EjTFL1-1* and *EjTFL1-2* during inflorescence development. **(F)** The phenotype of the inflorescence from September to December. The yellow background indicates the initiation period of flower bud differentiation, and the purple background represents the flower-opening period. The black arrow points to the floral meristem. The black circle points to the panicle of loquat. Error bars represent ± SE from three biological replicates.

We conducted year-round follow-up observation and sampling of the shoot apices and found obvious inflorescences from the end of August to the beginning of September (Figures 3A,F). To examine the time of floral bud differentiation, we prepared paraffin sections of the shoot apices in different periods and examined them by microscope. In late June, the growing point of the apical bud was flat (June 27, Figure 3B), indicating the vegetative growth period. Subsequently, the growing point was raised and a distinct floral meristem can be seen (July 11, Figure 3B), and the floral bud began to differentiate, marking the change from the vegetative stage to the reproductive stage. At the same time, *EjAP1-1* and *EjAP1-2* began to be expressed in the shoot apices in early July (Figure 3C), indicating that the floral bud began to differentiate. Therefore, we determined that the loquat flower bud differentiation of ‘Jiefangzhong’ loquat in Guangzhou occurs at the end of June and early July 2017.

To identify whether the expression levels of *EjTFL1s* changed in the shoot apices during different development stages, the qRT-PCR analysis was performed from May to December. The expression level of *EjTFL1-1* was the highest in May and then gradually decreased before floral bud differentiation, when almost no expression was observed (July 24th to November 10th) (Figure 3D). In contrast, *EjTFL1-2* expression was high in late May and decreased thereafter until reaching a minimum level in early September (Figure 3D). Interestingly, we found that the *EjFTL1s* started to be expressed again during flower-opening period (Figure 3E). In summary, *EjTFL1s* showed a relatively high expression level in the vegetative growth stage, and showed a downward trend after entering flower bud differentiation until no expression was found.

**FIGURE 4:**
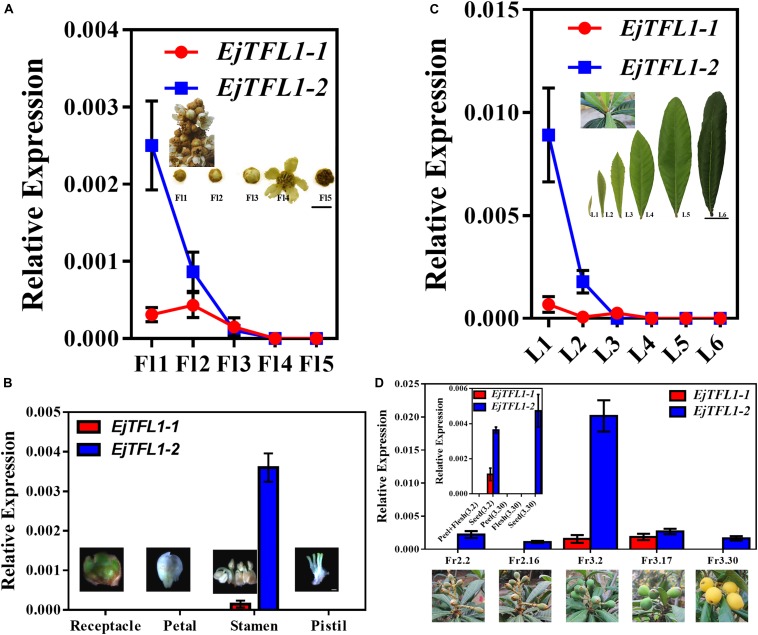
Expression patterns of *EjTFL1-1* and *EjTFL1-2* in leaves, flowers, and fruits of loquat. **(A)** Relative expression levels of *EjTFL1-1* and *EjTFL1-2* in flowers of different maturities in the same period (scale is 1 cm). **(B)** Relative expression levels of *EjTFL1-1* and *EjTFL1-2* in different tissues of flower buds (scale 2 mm). **(C)** Relative expression levels of *EjTFL1-1* and *EjTFL1-2* in leaves at different levels of maturity in the same period (scale is 5 cm). **(D)** Relative expression levels of *EjTFL1-1* and *EjTFL1-2* in fruit development (the small figure shows the relative expression levels of *EjTFL1-1* and *EjTFL1-2* in different tissues of fruits at different times). ‘Fl1’ indicate the stage 1 of flowers, ‘L1’ indicate the stage 1 of leaves, ‘Fr2.2’ indicates the fruit of February 2, and so on. Error bars represent ± SE from three biological replicates.

To further explore the function of *EjTFL1s*, we performed qRT-PCR analysis of different tissues of flowers and flowers at different stages of maturity in opening inflorescences (December). *EjTFL1-1* expression was extremely low in these tissues and organs or almost undetectable (Figures 4A,B). *EjTFL1-2* gradually decreased in Fl1-Fl3 and was almost undetectable in Fl3, Fl4, and Fl5, being expressed only in the stamen (Figures 4A,B).

Moreover, *EjTFL1-1* was slightly expressed in L1 and L3 and not expressed in the other periods in leaves at different stages of maturity in the same period (Figure 4C). *EjTFL1-2* was not expressed in L3–L6 but showed relatively high expression in young and tender leaves (L1, L2) (Figure 4C). Previous studies have shown that the L1–L2 period in leaves involves rapid cell proliferation (Gonzalez et al., 2012; Nelissen et al., 2016). Interestingly, flower (stamen) ripening occurs in Fl1-Fl3. These results suggested that *EjTFL1-2* may be involved in cell proliferation. Additionally, in the fruit, we found that *EjTFL1-1* and *EjTFL1-2* were mainly expressed during the fruit-expansion (cell expansion) (see Su et al., 2017) process (Fr3.2-Fr3.17) and were only expressed in seeds during the fruit development process (Figure 4D).

### Ectopic Expressions of *EjTFL1s* in *Arabidopsis*

To investigate the effects of the two *EjTFL1s* in regulating flowering time and inflorescence phenotypes, we overexpressed the *EjTFL1s* under control of the CaMV 35S promoter in wild-type Col-0 *Arabidopsis.* Nine transgenic plants were obtained for both groups (35S:*EjTFL1-1* and 35S:*EjTFL1-2*), and two T3 homozygous lines of each genotype were used for further investigation.

**FIGURE 5:**
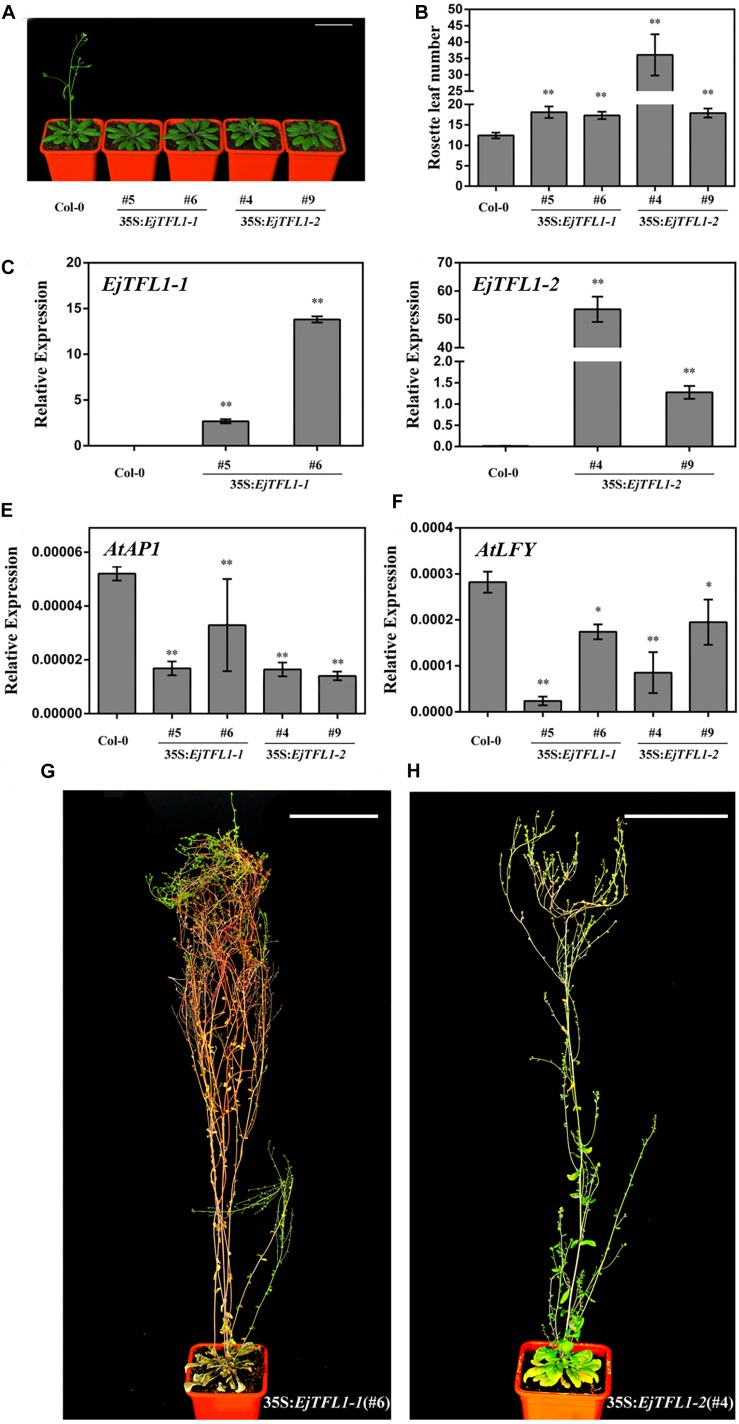
Ectopic expression of *EjTFL1s* in *Arabidopsis*. **(A)** Forty-day-old *35S:EjTFL1s* transgenic lines flowered later than did wild-type Col-0 plants. Scale bars = 5 cm. **(B)** Statistics of *35S:EjTFL1* transgenic lines and wild-type Col-0 rosette leaves (*n* ≥ 10). **(C,D)** Expression levels of *EjTFLs* in 40-day-old transgenic lines and wild-type Col-0 plants. **(E,F)** Expression levels of *AtAP1* and *AtLFY* in 40-day-old transgenic lines and wild-type Col-0 plants. **(G,H)** Phenotype of *35S:EjTFL1*-transgenic lines, the scale is 10 cm. The whole-plant was used for qRT-PCR. Asterisks indicate significant differences from Col-0, *p < 0.05, **p < 0.01, by Student’s *t*-test. Error bars indicate ± SE from 3 biological replicates.

Compared with wild-type Col-0 plants, the *35S:EjTFL1-1-* and *35S:EjTFL1-2-*transgenic plants displayed delayed flowering (Figure 5A). The *35S:EjTFL1-1-* and *35S:EjTFL1-2-*transgenic plants produced approximately 17-18 rosette leaves, and *35S:EjTFL1-2* line4 (#4) produced approximately 36 rosette leaves (Figure 5B). In contrast, wild-type Col-0 plants produced approximately 12–13 rosette leaves (Figure 5B). qRT-PCR was applied to examine whether the late-flowering phenotype was associated with *EjTFL1s* in transgenic plants. The expression levels of *EjTFL1s* were significantly increased in the transgenic plants (Figures 5C,D). In addition, the floral meristem identity genes *AtAP1* and *AtLFY* were clearly downregulated in different transgenic lines (Figures 5E,F). Interestingly, *35S:EjTFL1s* transgenic lines exhibited late flowering, and the stem lengths were much higher than those of wild-type Col-0 plants (Figures 5A,G,H).

In addition, overexpression of *EjTFL1s* in wild-type Col-0 caused morphological and structural changes. The transgenic plants displayed more inflorescence branches than did wild-type Col-0 (Supplementary Figures S2A–C), with 2–4 inflorescence branches in the latter but as many as 9–17 in the former (Supplementary Figure S3). Compared with wild-type Col-0 plants (Supplementary Figure S2D), the inflorescence phenotypes of some *35S:EjTFL1s*-transgenic lines were not significantly different (Supplementary Figures S2E,F). However, there were significant differences in the inflorescence phenotype of other transgenic lines compared to the wild-type Col-0 plants (Supplementary Figures S2G,H). As shown in Supplementary Figure S2G,H, a leaf-like organ appearance, similar to the phenotype described by Ferrandiz et al. (2000), was produced. Moreover, deformed siliques and abnormal cauline leaves were generated and easily distinguished these plants from wild-type Col-0 (Supplementary Figures S2I–L).

### GA_3_ and Short-Day Conditions Up-Regulated the Expression of *EjTFL1s* and Repressed Flowering

Studies in other woody fruit trees, such as apple (Zhang S. et al., 2016) mango (Nakagawa et al., 2012), and citrus (Goldberg-Moeller et al., 2013), have shown that GA_3_ treatment results in no or less flowering. And that, it is worth noting that loquat flower bud differentiation occurs during the longest period of sunshine in the year, that is, after the summer solstice (June 22), a time when the expression level of *EjTFL1s* decreased (Figure 3D). Therefore, we speculate that long-day conditions inhibit *EjTFL1s* expression, thereby promoting flower bud differentiation in loquat. To assess whether *EjTFL1s* are regulated by GA_3_ or photoperiod, we designed two experimental protocols (exogenous GA_3_ and short-day treatments).

**FIGURE 6:**
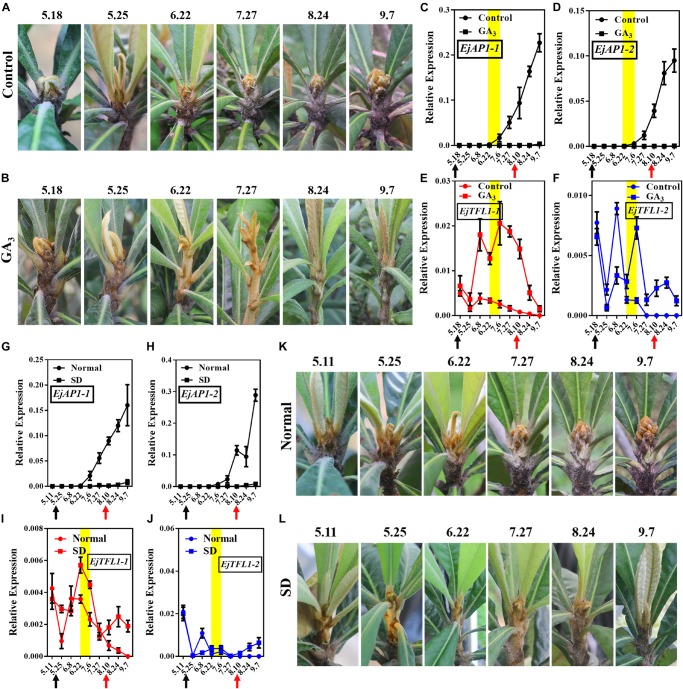
Effects of GA_3_ treatment and short-day treatment on flowering in loquat. **(A)** The shoot apices phenotype of the control group from May 18 to September 7. **(B)** The shoot apices phenotype of the gibberellin-treated group from May 18 to September 7. **(C–F)** Relative expression levels of *EjAP1s* and *EjTFL1s* in the shoot apices of the gibberellin treatment and control groups. **(G–J)** Relative expression levels of *EjAP1s* and *EjTFL1*s in the shoot apices of short-day-treated and normally grown plants. **(K)** The shoot apices phenotype of normal growth from May 11 to September 7. **(L)** The shoot apices phenotype of the short-day treatment from May 11 to September 7. The yellow background indicates the initiation period of flower bud differentiation. The two experimental treatment times were from 18th May to 10th August. The black arrow indicates the time at which the treatment started (18th May) and the red arrow indicates the time at which the treatment ended (10th August). Error bars represent ± SE from three biological replicates.

Interestingly, after exogenous GA_3_ and short-day treatments, the loquat plants were unable to undergo flower bud differentiation, though the control and normally grown plants did show flower bud differentiation (Figures 6A,B,K,L). We also found that the flower-specific genes *EjAP1-1* and *EjAP1-2* were not expressed after GA_3_ or short-day treatment (Figures 6C,D,G,H). Nonetheless, after the release of treatment (August 10th), the *EjAP1s* showed a lower level of expression in early September (Figures 6C,G,H). Combining phenotypic observations and qRT-PCR results, we found that GA_3_ and short-day treatments inhibit flower bud differentiation in loquat.

Importantly, *EjTFL1-1* and *EjTFL1-2* were abundantly expressed after GA_3_ treatment and maintained high expression level at the end of June, a time when the control group was undergoing flower bud differentiation and *EjTFL1-1* and *EjTFL1-2* begin to gradually decrease until they are not expressed (Figures 6E,F). In the short-day treatment group, we found that the change in *EjTFL1-1* expression after treatment was not as significant as that after GA_3_ treatment; however, from July 6 to September 7, we still observed a relatively high expression level. The expression level of *EjTFL1-2* was not significantly increased at the beginning of the short-day treatment, but a relatively high level of expression began after July 27.

In summary, GA_3_ clearly promoted *EjTFL1-1* expression, while SD treatment may promote *EjTFL1-1* expression with a delay, but it does not show clear results on *EjTFL1-2* after these treatments.

**FIGURE 7:**
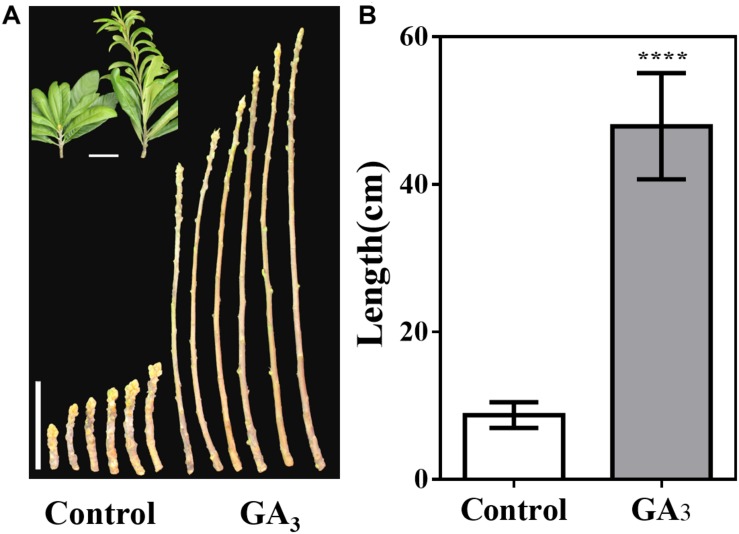
GA_3_-treatment promotes stem growth. **(A)** Stem phenotype of GA_3_-treated and control plants. **(B)** Stem length statistics of GA_3_-treated and control plants (*n* = 30). The GA_3_-treatment times were from 18th May to 10th August. Asterisks indicate significant differences from Control, ****p < 0.0001, by Student’s *t*-test. Error bars represent the standard deviation from *n* = 30.

Furthermore, the stem tip showed strong vegetative growth after GA_3_ treatment, and we calculated the stem length of the control group and the GA_3_ treatment group from September 7. The average stem length of the control group was 8.7 cm, while average stem length of the GA_3_ treatment group was 47.9 cm (Figures 7A,B).

### Cloning and Analysis of *EjTFL1* Promoters

To investigate the potential reasons leading to the difference in temporal and spatial expression of *EjTFL1-1* and *EjTFL1-2* during the growth and development of loquat and the difference in response to GA_3_ and short-day treatment, we searched through the whole genome information of loquat and found that the structural elements of the promoter region of *EjTFL1-1* and *EjTFL1-2* were significantly different in the 2 kbp upstream regions of the start codon of the *EjTFL1* genes. So, we cloned the 2169 bp promoter region upstream of the *EjTFL1-1* initiation codon ATG and the 2000 bp promoter region upstream of the *EjTFL1-2* initiation codon ATG (see Supplementary Sequences).

Both of these promoter sequences contain a large number of CAAT-boxes associated with transcription initiation and TATA-box promoter core elements of the eukaryotic RNA polymerase II recognition site. Both also contain the following: photoresponsive elements, Box 4, GATA-motif, AE-box and GT1-motif; jasmonic acid (JA) response elements TGACG-motif and CGTCA-motif; and antioxidant response elements (AREs) (Table 1). In addition, the *EjTFL1-1* promoter contains the photoresponsive elements TCCC-motif, LAMP-element, MRE and Sp1, as well as the auxin response element TGA-element and salicylic acid (SA) response element TCA-element (Table 1). The *EjTFL1-2* promoter contains the photoresponsive element ACE, G-Box, GA-motif, I-box, and TCT-motif, as well as the abscisic acid (ABA) response element (ABRE) and the circadian rhythm regulatory element (Table 1). Importantly, a gibberellin response element, TATC-box, is present in the *EjTFL1-1* promoter, and a *cis*-acting regulatory element, CAT-box, related to meristem expression is present in the *EjTFL1-2* promoter (Table 1).

**TABLE 1:** Prediction of *EjTFL1-1* and *EjTFL1-2* promoter *cis*-acting elements.

Element name	Sequence	Number of elements of the *EjTFL1-1-pro*	Number of elements of the *EjTFL1-2-pro*	Function
Box 4	ATTAAT	6	6	Part of a conserved DNA module involved in light responsiveness
GATA-motif	AAGATAAGATT	1	1	Part of a light responsive element
AE-box	AGAAACTT	1	1	Part of a module for light response
GT1-motif	GGTTAAT	2	1	Light responsive element
ACE	CTAACGTATT	0	1	*Cis*-acting element involved in light responsiveness
G-Box	CACGTG	0	1	*Cis*-acting regulatory element involved in light responsiveness
GA-motif	ATAGATAA	0	2	Part of a light responsive element
I-box	atGATAAGGTC	0	1	Part of a light responsive element
TCT-motif	TCTTAC	0	1	Part of a light responsive element
TCCC-motif	TCTCCCT	1	0	Part of a light responsive element
LAMP-element	CTTTATCA	1	0	Part of a light responsive element
MRE	AACCTAA	1	0	MYB binding site involved in light responsiveness
Sp1	GGGCGG	1	0	Light responsive element
TATC-box	TATCCCA	1	0	*Cis*-acting element involved in gibberellin-responsiveness
CAT-box	GCCACT	0	1	*Cis*-acting regulatory element related to meristem expression
CGTCA-motif	CGTCA	3	3	*Cis*-acting regulatory element involved in the MeJA-responsiveness
TGACG-motif	TGACG	3	3	*Cis*-acting regulatory element involved in the MeJA-responsiveness
TCA-element	TCAGAAGAGG	1	0	*Cis*-acting element involved in salicylic acid responsiveness
ABRE	CACGTG	0	1	*Cis*-acting element involved in the abscisic acid responsiveness
TGA-element	AACGAC	1	0	Auxin-responsive element
ARE	AAACCA	3	1	*Cis*-acting regulatory element essential for the anaerobic induction
Circadian	CAAAGATATC	0	1	*Cis*-acting regulatory element involved in circadian control

### Subcellular Localization and Interaction of EjTFL1s and EjFD

In *Arabidopsis*, TFL1 forms a complex with FD and inhibits flowering by antagonizing the activity of FT-FD complexes (Abe et al., 2005; Hanano and Goto, 2011). Similar results have been reported in kiwifruit (Varkonyi-Gasic et al., 2013), rose (Randoux et al., 2014), and chrysanthemum (Higuchi and Hisamatsu, 2015). We isolated one *FD* homologous gene from loquat, *EjFD* (see Supplementary Sequences), and phylogenetic analysis indicated that EjFD shares high identity with FD in other plants, especially those of the Rosaceae family, such as 98, 74, and 75% identity with EdFD1, EdFD2, and MdFD, respectively (Supplementary Figure S1A). Alignment of deduced amino acid sequences revealed the presence of a conserved bZIP domain (Supplementary Figure S1B).

**FIGURE 8:**
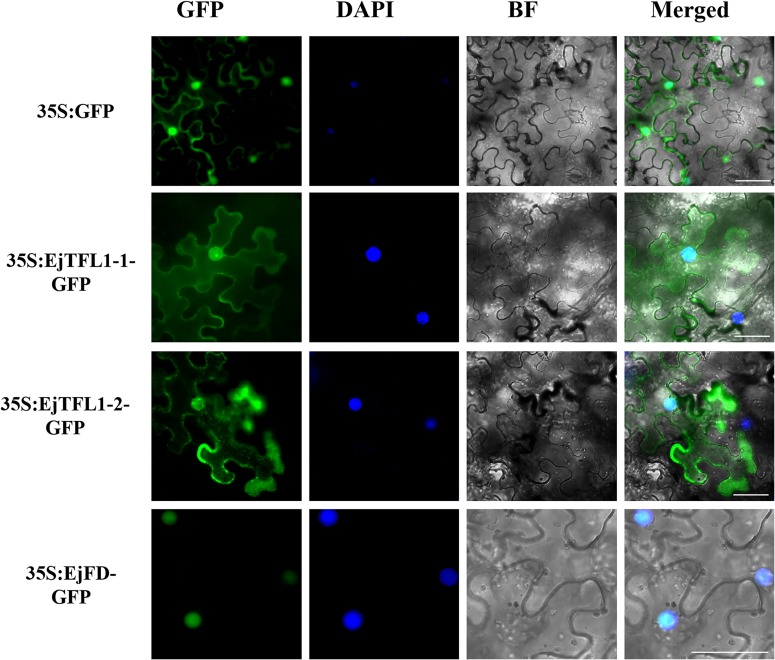
Subcellular localization of EjTFL1s and EjFD. 35S-EjTFL1s-GFP and 35S-EjFD-GFP were transiently transformed into *N. benthamiana* leaves. GFP, GFP fluorescence; DAPI(4,6-diamidino-2-phenylindole), staining indicates nuclear localization; BF, bright field; Merged, merged image of GFP, BF, and DAPI. Scale bars = 50 μm. 35S: GFP as a control.

To determine the subcellular localization of EjTFL1s and EjFD, we constructed plasmids for expressing 35S:EjTFL1-1-GFP, 35S:EjTFL1-2-GFP and 35S:EjFD-GFP fusion proteins and transiently expressed them in *N. benthamiana* leaves. GFP signals in the nucleus and cytoplasm were found for the two EjTFL1-GFP fusion proteins, whereas the EjFD-GFP fusion protein was only detected in the nucleus (Figure 8). These results showed that both EjTFL1-1 and EjTFL1-2 localized to the cytoplasm and nucleus but that EjFD localized only to the nucleus.

**FIGURE 9:**
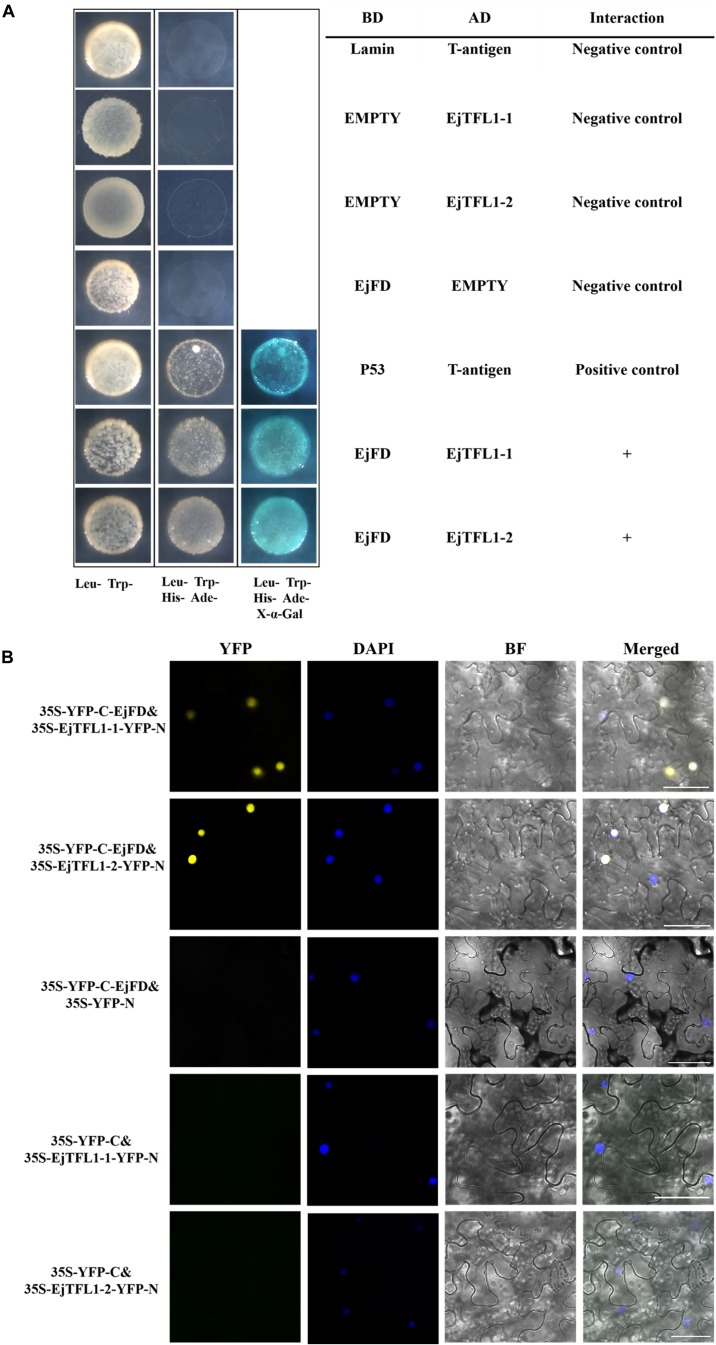
EjTFL1s interacts with EjFD. **(A)** Y2H analysis of the interaction of EjTFL1s and EjFD in yeast cells. pGBKT7-EjFD is a fusion bait protein, pGADT7-EjTFL1-1 and pGADT7-EjTFL1-2 are fusion prey proteins, pGBKT7-Lam + pGADT7-T, pGBKT7 + pGADT7-EjTFL1s, and pGBKT7-EjFD + pGADT7-T are negative controls, pGBKT7-53 + pGADT7-T are positive controls. The yeast cells co-transfused with the plasmid can grow in the selective medium lacking leucine (Leu-), tryptophan (Trp-), histidine (His-), and adenine (Ade-) but contain 600 μM Aureobasidin A, and turn blue in the presence of X-*α*-Gal (20 mg/mL), such yeast cells are identified as positive interactions. **(B)** BiFC system analysis of the interaction of EjTFL1s and EjFD in *N. benthamiana* leaf epidermal cells. The 35S-YFP-C-EjFD + 35S-EjTFL1s-YFP-N, 35S-YFP-C-EjFD + 35S-YFP-N, 35S-YFP-C + 35S-EjTFL1s-YFP-N were transiently transformed into *N. benthamiana* leaves. YFP, YFP fluorescence; DAPI (4,6-diamidino-2-phenylindole) staining indicates nuclear localization; BF, bright field; Merged, combined YFP, BF and DAPI images; 35S-YFP-C-EjFD + 35S-YFP-N and 35S-YFP-C + 35S-EjTFL1s-YFP-N as negative control. Scale bar = 50 μm.

To evaluate whether EjTFL1s and EjFD can interact, we performed Y2H and BiFC assay. The yeast cells co-transformed with AD-EjTFL1s and BD-EjFD were able to grow on selective medium (Leu-, Trp-, His-, Ade-) and appeared blue after X-α-Gal treatment (Figure 9A). Thus, EjTFL1s can interact with EjFD in yeast cells. In our BiFC experiment, a yellow fluorescent signal in the nucleus was detected in *N. benthamiana* leaf epidermal cells co-expressing EjTFL1s and EjFD, with no fluorescent signal observed for the control group (Figure 9B). These results confirmed that EjTFL1s can interact with EjFD in *N. benthamiana* cells.

In summary, EjTFL1-1 and EjTFL1-2 proteins were able to interact with EjFD proteins, suggesting that EjTFL1s may inhibit floral bud differentiation in loquat by interacting with EjFD, which is the same as the *AtTFL1* regulation pattern.

### The EjTFL1-EjFD Complex Inhibits *EjAP1-1* Promoter Activity

**FIGURE 10:**
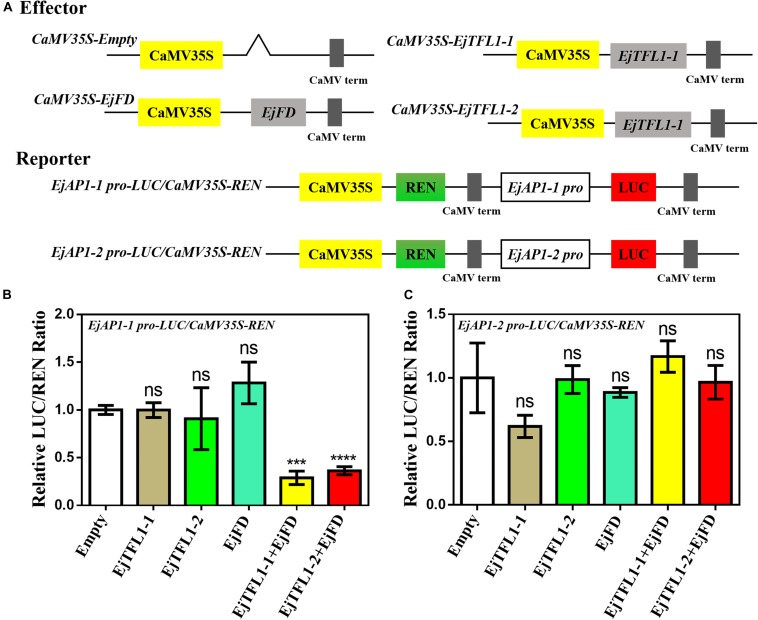
A dual luciferase reporter assay validated the transcriptional activation of *EjAP1* promoters by EjTFL1s, EjFD, and EjTFL1s-EjFD. **(A)** Schematic diagram of effector and reporter plasmid construction. **(B)** EjTFL1s, EjFD, and EjTFL1s-EjFD act on the *EjAP1-1* promoter. **(C)** EjTFL1s, EjFD, and EjTFL1s-EjFD act on the *EjAP1-2* promoter. The activation capabilities of EjTFL1s and EjFD were calculated by the ratio of LUC to REN. The ratio of empty vector LUC/REN was used as a control and set as 1. Error bars represent ± SE from three biological replicates. Asterisks indicate significantly different values compared with those for the empty vector (calculated by Student’s *t*-test), 0.0001 < *** *p* < 0.001; ****p < 0.0001; ns, indicates that the difference was not significant.

*AtAP1* is downregulated in transgenic lines, and the expression trends of *EjTFL1s* and *EjAP1s* are opposite during flower bud differentiation. We hypothesized that *EjTFL1s* may also regulate flower bud differentiation by inhibiting expression of *EjAP1s* in loquat. Thus, we cloned *EjAP1-1* and *EjAP1-2* gene promoter regions [1761 bp (*EjAP1-1*) and 2001 bp (*EjAP1-2*) upstream of the start codon ATG, see Supplementary Sequences] and tested whether EjTFL1s or EjTFL1-EjFD complex activated or repressed the expression of the *EjAP1s* using a dual luciferase reporter system (see Figure 10A for the details of vector construction).

The results showed that EjTFL1s and EjFD had no obvious inhibitory effect on the *EjAP1* promoters and that the EjTFL1-EjFD complex had no significant inhibitory effect on the *EjAP1-2* promoter (Figures 10B,C). Importantly, the EjTFL1-1-EjFD complex and EjTFL1-2-EjFD complex significantly repressed the promoter activity of *EjAP1-1*, and the LUC/REN ratio was 0.29-fold and 0.36-fold that of the empty vector, respectively (Figure 10B). These results suggest that the interaction between EjTFL1s and EjFD inhibits flower bud differentiation and maintains vegetative growth in loquat by inhibiting expression of *EjAP1-1*.

## Discussion

Different from Rosaceae relatives such as apple or pear (flower bud differentiation in summer or autumn, flowering in the next spring), cultivated loquat flower bud differentiation occur in summer, followed by blossoming from late fall into winter and fruits ripening from late spring into summer (Lin, 2007). As there are few other fresh fruits in late spring and early summer, the loquat just complements the fruit requirement in the market at this time. Moreover, various *Eriobotrya* species, such as *Eriobotrya deflexa* Nakai, blossom in spring as do apple and pear (Gu and Spongberg, 2003). The changes in the flowering period of *Eriobotrya* species suggest that these plants have undergone some unknown changes during evolution. Therefore, it is meaningful to explore the flower-regulating network of loquat, which may also provide some references for the study of flowering regulation in other fruit trees in Rosaceae.

In *Arabidopsis*, *TFL1* act as floral inhibitor (Bradley et al., 1997; Yoo et al., 2010; Hanano and Goto, 2011; Huang et al., 2012). Similarly, in this study, our results indicated that the structure and functions of flowering regulation of *EjTFL1s* are relatively conserved. *EjTFL1s* might be mainly involved in vegetative growth and inhibition of in flower bud differentiation. However, *EjTFL1-1* and *EjTFL1-2* have some different functions during the growth and development of loquat. For example, their expression patterns are different in roots and fruits. (Figures 2, 4D). Furthermore, the expression levels in the same tissue of the same period were much different (Figures 2–4). Interestingly, *PopCEN1* and *PopCEN2*, two *TFL1* homologs in *populus*, also have different tissue-type expression patterns and exhibited relatively conserved functions in flowering (Mohamed et al., 2010). Similarly, *EjTFL1-1* and *EjTFL1-2* may also have functional differences or functional redundancy, ensuring the normal growth and development of loquat. Changes in the function of homologous genes in plants may play a positive role in the response to sophisticated exogenous and endogenous environments during growth and development.

In the study of *Eriobotrya deflexa* Nakai forma *koshunensis*, it was found that EdFT can interact with EdFDs, which may promote flower bud differentiation in loquat (Zhang L. et al., 2016). In *Arabidopsis*, TFL1 forms a complex with FD and inhibits flowering by antagonizing the activity of FT-FD complexes, and thereby inhibit the expression of downstream AP1 (Abe et al., 2005). AP1 was reported to bind to at least two sites in the 3′ region of *TFL1* to inhibit its expression (Kaufmann et al., 2010). In this study, *EjTFL1s* was able to interact with EjFD, and the expression trends of *EjTFL1s* and *EjAP1s* were completely opposite (Figures 3C,D); In addition, the EjTFL1-EjFD complex significantly repressed the promoter activity of *EjAP1-1* (Figure 10).

We speculated that the regulatory network of *EjTFL1s* in the loquat should be as follow: *EjTFL1s* show relatively high expression in the vegetative growth stage (Figure 3D), and EjTFL1s and EjFD form a complex (Figure 9) to inhibit expression of *EjAP1-1*, thereby suppressing flower bud differentiation and promoting vegetative growth. Additionally, *EjTFL1s* integrate photoperiod signal. (Figures 3D, 6I,J), and *EjAP1s* begin to be abundantly expressed in long-days (Figure 3D). *EjAP1s* regulates expression of *EjTFL1s* through negative feedback, promoting a rapid decrease in *EjTFL1s* expression and at the same time relieving inhibition of *EjAP1-1* by EjTFL1s and accelerating flower bud differentiation. This type of feedback mechanism promotes flowering transformation and allows plants to reproduce with high efficiency.

It has been found that *TFL1* homologues in different plants are relatively conserved in function, which promotes vegetative growth and delays flowering, such as in *Arabidopsis* (Hanano and Goto, 2011), rice (Nakagawa et al., 2002), chrysanthemum (Higuchi and Hisamatsu, 2015), dogwood (Liu et al., 2016), apple (Flachowsky et al., 2012), black cherry (Wang and Pijut, 2013), and pomegranate (Patil et al., 2018). Furthermore, ectopic expression of *EjTFL1s* in *Arabidopsis* has multiple effects, including modified plant height, branching, and inflorescence, silique and cauline leaf morphology (Figures 5G,H and Supplementary Figure S3). For the *TFL1* homolog *SP (SELF-PRUNING)* in tomato, *sp.* mutations induce sympodial prolongation and decreased internode numbers (Pnueli et al., 1998). Overexpression of *RCN1* or *RCN2* and rice *TFL1/CEN* homologues produced more branches and denser panicle morphology (Nakagawa et al., 2002), and the *Cornus TFL1* homolog controls inflorescence development (Liu et al., 2016). These findings suggest that *TFL1*-like genes control inflorescence architecture in different species.

The differences in the *cis*-acting elements of the *EjTFL1-1* and *EjTFL1-2* promoters may be responsible for their different patterns of their response to GA_3_. In our results, *EjTFL1-1* responded to the GA_3_ signal more rapidly and strongly than did *EjTFL1-2* (Figures 6E,F), and the *EjTFL1-1* promoter contains a gibberellin signal element, suggesting that *EjTFL1-1* might directly respond to the GA_3_ signal and *EjTFL1-2* may respond to the GA_3_ signal through another pathway. Studies have shown that GA regulates cell expansion and division primarily by eliminating inhibition by DELLA repressors (Yang et al., 1996; Cheng et al., 2004; Ubeda-Tomas et al., 2008; Lee et al., 2012). In this study, the transcription level of *EjTFL1s* increased significantly after GA_3_ treatment (Figures 6E,F), and loquat plants showed vigorous vegetative growth (Figures 6B, 7A). The expression trend illustrated in Figure 4 indicates that *EjTFL1s* are mainly expressed in young tissues. Additionally, *Arabidopsis 35S:EjTFL1*-transgenic lines displayed significantly increased stem length and delayed flowering. All this evidence indicates that *EjTFL1-1* and *EjTFL1-2* may play a very important role in the vegetative growth process. Both the previous qRT-PCR analysis and the results of promoter analysis suggest that GA_3_ regulates the vegetative growth of loquat by modulating the expression of EjTFL1s. The results of this study provide some references for analyses of other woody fruit trees and also provide a new idea for the selection of fast-growing tree species.

Moreover, the *EjTFL1-1* and *EjTFL1-2* promoters contain a large number of photoresponsive elements, and their expression is affected by SD-treatment (Figures 6I,J), which may be regulated by photoperiod. Interestingly, in short-day *Fragaria vesca*, short days suppressed *FvTFL1* expression and long-day conditions activated it (Koskela et al., 2012). However, in our study, qRT-PCR results showed that *EjTFL1s* were inhibited under long-day conditions and increased under short-day conditions (Figures 3D, 6I,J). This difference provides an important clue for us to further explore the unique flowering habits of loquat. The *EjTFL1-2* promoter contains the CAT-box, a regulatory component involved in meristem expression, and this result suggesting that *EjTFL1-2* may be involved in cell proliferation. Importantly, Koskela et al. (2017) found that a deletion located 2547 bp upstream of the transcription start site of *FvTFL1* have consequences on expression and function of *FvTFL1*. This suggested that further away from the transcription start site of the *EjTFL1s* may also contain more structural elements that affect its function and expression.

## Conclusion

*EjTFL1-1* and *EjTFL1-2* possess conserved amino acids and are mainly expressed in young tissues. Furthermore, the EjTFL1s-EjFD complex significantly repressed the promoter activity of *EjAP1-1*. Ectopic overexpression of the two *EjTFL1s* in wild-type *Arabidopsis* Col-0 delayed flowering time. Our findings suggest that *EjTFL1-1* and *EjTFL1-2* inhibit flower bud differentiation and promote vegetative growth in loquat by integrating GA_3_ and photoperiod signals. These findings contribute to our understanding of the molecular mechanism of loquat flowering time regulation and may have wider implications for future manipulation of the time of flowering or bearing fruit.

## Data Availability Statement

All datasets generated for this study are included in the article/Supplementary Material.

## Author Contributions

YJ, YZ, YG, and SL conceived and designed the research. YJ and YZ mainly performed the research and wrote the manuscript. LZ, WS, and JP finished some parts of the experiments. HS and XY supervised the research. YG and SL revised and approved the manuscript.

## Conflict of Interest

The authors declare that the research was conducted in the absence of any commercial or financial relationships that could be construed as a potential conflict of interest.

## Footnotes

1 http://bioinformatics.psb.ugent.be/webtools/plantcare/html/
