# Supplementary material for: EjTFL1 Genes Promote Growth but Inhibit Flower Bud Differentiation in Loquat
Source: Front Plant Sci. 2020 May 15;11:576. doi: 10.3389/fpls.2020.00576 (PMC7247538; doi:10.3389/fpls.2020.00576)
Supplement: Supplementary file 1 [file Data_Sheet_1.docx]

***EjTFL1* promote growth but inhibit flower bud differentiation in loquat**

## Supplementary Table

Table S1. PCR primers used for cloning

| Name | Forward primer (5’-3’) | Reverse primer (5’-3’) |
| --- | --- | --- |
| *EjTFL1-1* | ATGACAAGAGCCTTGGAGCCT | CTAGCGTCTTCTAGCTGCAGT |
| *EjTFL1-2* | ATGGCAAGAATCCCGGAGC | CTAGCGTCTTCTAGCTGCAGT |
| *EjFD* | ATGTTGTCATCAACAGGTAG | TCAAAATGGAGCTGTTGAT |
| *EjAP1-1-pro* | GACGGCGAAGATGTAAAGCCATACTT | GATTAAATACTTATGGACTCTCAGT |
| *EjAP1-2-pro* | AAAGGGGGGATATAGACCTTACCTC | TGATTAAATATTAATGGACTCTCAGT |
| *EjTFL1-1-pro* | CTAGCAGCTTGGCAGCAGGCACTCAG | TTTATGAGAGAGAGAGAGAGAGAGA |
| *EjTFL1-2-pro* | ACGTGCCCACGAGGTGAGGGGGGAG | TTTAAGAGAAGAGAGTAGTACTTCTT |

Table S2. Primers used for the analysis of gene expression by qPCR in loquat

| Name | Forward primer (5’-3’) | Reverse primer (5’-3’) |
| --- | --- | --- |
| *Ejβ-actin* | GGATTTGCTGGTGATGATGC | CCGTGCTCAATGGGATACTT |
| *EjTFL1-1* | CTGTCGCTGCCGTCTACTTC | TGTGGATAATGGATGGAGGAG |
| *EjTFL1-2* | CTGCAGCTAGAAGACGCTAG | GTACGTAATAGTGGCCTAATGG |

Table S3. Primers used for the analysis of gene expression by qPCR in Arabidopsis

| Name | Forward primer (5’-3’) | Reverse primer (5’-3’) |
| --- | --- | --- |
| *TUB2* | ATCCGTGAAGAGTACCCAGAT | AAGAACCATGCACTCATCAGC |
| *AP1* | CATGGGTGGTCTGTATCAAGAAGAT | CATGCGGCGAAGCAGCCAAGGTT |
| *LFY* | ACGCCGTCATTTGCTACTCT | CTTTCTCCGTCTCTGCTGCT |
| *EjTFL1-1* | CATTTGGAAGAGAGGTGGTG | CGAGGAAGGTGTGTTGATTG |
| *EjTFL1-2* | CCTTCACTCCAACAACACACA | TCTGGGTCTATCATCACCAAAG |

Table S4. PCR primers used for vector construction

| Vector Name | Primer name | Primer sequence (5'-3') |
| --- | --- | --- |
| *EjTFL1-1-*pGreen-*35S/-GPF* | *35S/GFP-TFL1-1-F* | gtcgacggtatcgataagcttATGACAAGAGCCTTGGAGCCT |
|  | *35S/GFP-TFL1-1-R* | tcccccgggctgcaggaattcGCGTCTTCTAGCTGCAGT |
| *EjTFL1-2-*pGreen-*35S/-GPF* | *35S/GFP-TFL1-2-F* | gtcgacggtatcgataagcttATGGCAAGAATCCCGGAGC |
|  | *35S/GFP-TFL1-2-R* | tcccccgggctgcaggaattcGCGTCTTCTAGCTGCAGT |
| *EjFD-GFP* | *GFP-FD-F* | gtcgacggtatcgataagcttATGTTGTCATCAACAGGTAG |
|  | *GFP-FD-R* | tcccccgggctgcaggaattcAAATGGAGCTGTTGAT |
| *EjTFL1-1- YFP-N* | *YFP-TFL1-1-F* | tcagatctcgagctcaagcttATGACAAGAGCCTTGGAGCCT |
|  | *YFP-TFL1-1-R* | gactctagatcaggtggatccGCGTCTTCTAGCTGCAGT |
| *EjTFL1-2- YFP-N* | *YFP-TFL1-2-F* | tcagatctcgagctcaagcttATGGCAAGAATCCCGGAGC |
|  | *YFP-TFL1-2-R* | gactctagatcaggtggatccGCGTCTTCTAGCTGCAGT |
| *YFP-C-EjFD* | *YFP-FD-F* | agagatctcgagctcaagcttATGTTGTCATCAACAGGTAG |
|  | *YFP-FD-R* | gctgcacgctgcccaggatccAAATGGAGCTGTTGAT |
| *EjTFL1-1-AD* | *AD-TFL1-1-F* | gccatggaggccagtgaattcATGACAAGAGCCTTGGAGCCT |
|  | *AD-TFL1-1-R* | cagctcgagctcgatggatccGCGTCTTCTAGCTGCAGT |
| *EjTFL1-2-AD* | *AD-TFL1-2-F* | gccatggaggccagtgaattcATGGCAAGAATCCCGGAGC |
|  | *AD-TFL1-2-R* | cagctcgagctcgatggatccGCGTCTTCTAGCTGCAGT |
| *EjFD-BD* | *BD-FD-F* | atggccatggaggccgaattcATGTTGTCATCAACAGGTAG |
|  | *BD-FD-R* | ctagttatgcggccgctgcagAAATGGAGCTGTTGAT |
| *62-SK-EjTFL1-1* | *SK-EjCO-FP* | cgctctagaactagtggatccATGACAAGAGCCTTGGAGCCT |
|  | *SK-EjCO-RP* | gtcgacggtatcgataagcttCTAGCGTCTTCTAGCTGCAGT |
| *62-SK-EjTFL1-2* | *SK-EjFT2-FP* | cgctctagaactagtggatccATGGCAAGAATCCCGGAGC |
|  | *SK-EjFT2-RP* | gtcgacggtatcgataagcttCTAGCGTCTTCTAGCTGCAGT |
| *62-SK-EjFD* | *SK-EjFD-FP* | cgctctagaactagtggatccATGTTGTCATCAACAGG |
|  | *SK-EjFD-RP* | gtcgacggtatcgataagcttTCAAAATGGAGCTGTTG |
| *LUC-EjAP1-1-pro* | *LUC-EjAP1-1-FP* | gtcgacggtatcgataagcttGACGGCGAAGATGTAAAGCCATAC |
|  | *LUC-EjAP1-1-RP* | cgctctagaactagtggatccGATTAAATACTTATGGACTCTCAG |
| *LUC-EjAP1-2-pro* | *LUC-EjAP1-2-FP* | gtcgacggtatcgataagcttAAAGGGGGGATATAGACCTTACCTC |
|  | *LUC-EjAP1-2-RP* | cgctctagaactagtggatccTGATTAAATATTAATGGACTCTCAG |

## Supplementary Figure


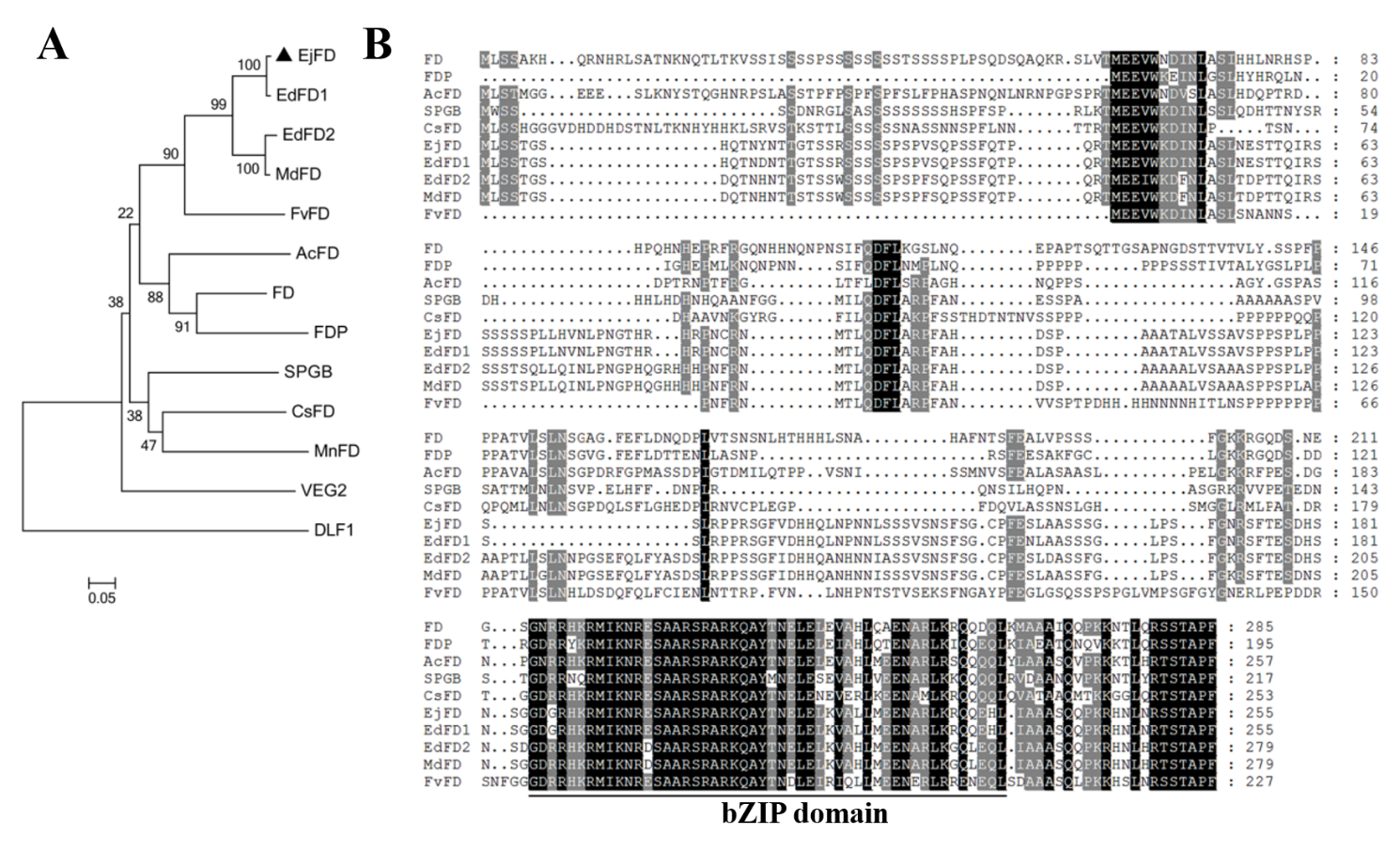


Figure S1. Sequence alignment and phylogenetic analysis of EjFD. (a) Phylogenetic analysis of FD proteins. Accession ID: EdFD1 (AMB72868), EdFD2 (AMB72869), FD (NP_195315), FDP (NP_001189545), AcFD (AGK89941), SPGB (ABL84199), MnFD (XP_010113304), SVEG2 (AKB91470), CsFD (BAN89468), DLF1 (NP_001105962), MdFD (XP_008337982), FvFD (XP_004289074). (B) Amino acid sequence alignment of FD-like genes from different species showed in (A).


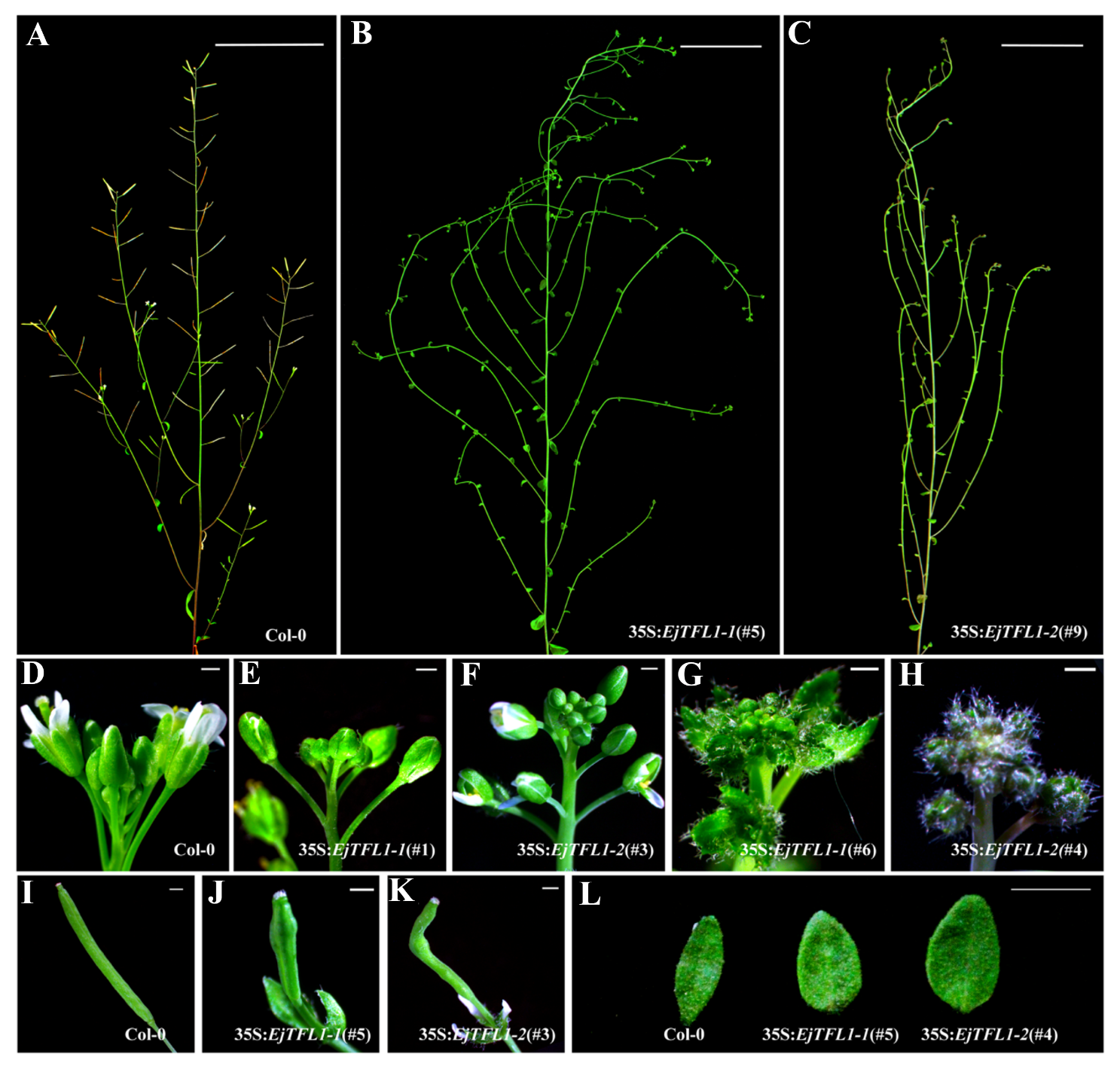


Figure S2. Phenotypes of *35S:EjTFL1*-transgenic lines and wild-type Col-0. (a)-(c) Inflorescence branching phenotypes of *35S:EjTFL1*-transgenic lines and wild-type Col-0. Scale bars=5 cm. (d)-(h) Inflorescence apical phenotypes of *35S:EjTFL1*-transgenic lines and wild-type Col-0. Scale bars=1 mm. (i)-(k) Silique phenotypes of *35S:EjTFL1*-transgenic lines and wild-type Col-0. Scale bars=1 mm. (l) Stem leaf phenotypes of *35S:EjTFL1*-transgenic lines and wild-type Col-0. Scale bars=1 cm.


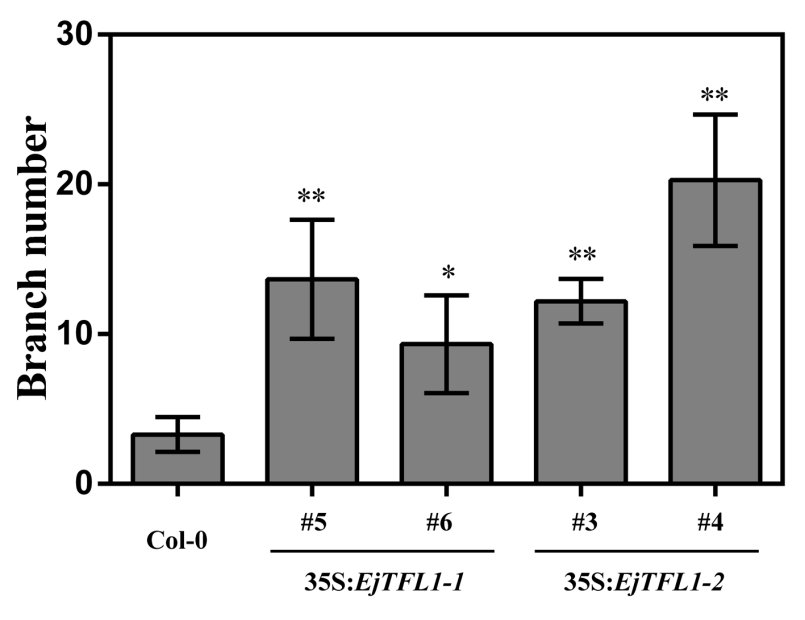


Figure S3. Comparison of branch numbers in transgenic and wild-type Col-0. Asterisks indicate significant differences between Col-0 and transgenic plants. * indicates *p*<0.05 and ** indicates *p*<0.01 in the t-test. n≥5.

## Supplementary Sequences

（1）*EjTFL1-1*

**1 ATGACAAGAGCCTTGGAGCCTCTGGTTGTTGGGAGAGTGATAGGAGATGTTCTTGATTCCTTCACTGCAACAACAAAAATGTCTGTCACTTACAAC**

**1 M T R A L E P L V V G R V I G D V L D S F T A T T K M S V T Y N**

**97 ACCAAGCTAGTCTGCAATGGACTTGAGCTCTTTCCTTCTGTTGTCACAGCCAAACCTAGGGTTGAGATTCAAGGAGGGGATATGAGATCTTTCTTT**

**33 T K L V C N G L E L F P S V V T A K P R V E I Q G G D M R S F F**

**193 ACTTTGGTGATGACAGACCCAGATTTTCCTGGCCCTAGTGATCCTTATCTAAGGGAGCACCTGCACTGGATTGTGACAGACATTCCAGGCACCACA**

**65 T L V M T D P D F P G P S D P Y L R E H L H W I V T D I P G T T**

**289 GATGCCACATTTGGAAGAGAGGTGGTGAGTTATGAGATGCCGAGGCCCAACATTGGCATCCACAGGTTTGTGTTTGTTCTCTTCAAGCAGAATCGA**

**97 D A T F G R E V V S Y E M P R P N I G I H R F V F V L F K Q N R**

**385 AGACAATCAATCAACACACCTTCCTCGAGGGATCACTTCAGCACTCGAAGCTTCGCGGCTGAAAATGACCTGGGTCTTCCTGTCGCTGCCGTCTAC**

**129 R Q S I N T P S S R D H F S T R S F A A E N D L G L P V A A V Y**

**481 TTCAACGCGCAGAGAGAAACTGCAGCTAGAAGACGCTAG**

**161 F N A Q R E T A A R R R ***

（2）*EjTFL1-2*

**1 ATGGCAAGAATCCCGGAGCCTCTAGTTGTTGGGAGAGTGATAGGAGATGTTCTTGATTCCTTCACTCCAACAACACACATGTCTGTCACTTACAAC**

**1 M A R I P E P L V V G R V I G D V L D S F T P T T H M S V T Y N**

**97 GCCAAGCTAGTCTGCAATGGACTTGAGCTCTTTCCTTCGGTTGTCACAGCCAAACCTAGAGTTGAGATTCAAGGAGGGGAGTTGAGATCTTTCTTT**

**33 A K L V C N G L E L F P S V V T A K P R V E I Q G G E L R S F F**

**193 ACTTTGGTGATGATAGACCCAGATTGTCCTGGCCCTAGTGACCCTTATCTAAGGGAGCACCTGCACTGGATTGTGACAGACATTCCAGGCACCACA**

**65 T L V M I D P D C P G P S D P Y L R E H L H W I V T D I P G T T**

**289 GATGCCGCATTTGGAAGAGAGGCGCTGAGCTATGAGATGCCAAGGCCCAACATTGGCATCCACAGGTTTGTGTTTGTTCTCTTCAAGCAGAAACGA**

**97 D A A F G R E A L S Y E M P R P N I G I H R F V F V L F K Q K R**

**385 AGACAATCAATCAACCCACCTTCCTCAAGGGATTGCTTCAGCACTCGAAGCTTCGCGGCTGAAAATGACCTGGGTCTTCCTGTCGCTGCCGTTTAC**

**129 R Q S I N P P S S R D C F S T R S F A A E N D L G L P V A A V Y**

**481 TTCAATGCGCAGAGAGAAACTGCAGCTAGAAGACGCTAG**

**161 F N A Q R E T A A R R R ***

（3）*EjFD*

**1 ATGTTGTCATCAACAGGTAGTCACCAAACCAACTACAACACAACCGGTACTTCATCCAGGTCCTCCTCCTCATCCCCATCACCAGTTTCACAACCC**

**1 M L S S T G S H Q T N Y N T T G T S S R S S S S S P S P V S Q P**

**97 TCCTCCTTCCAAACCCCACAAAGAACCATGGAAGAGGTTTGGAAAGACATCAATCTCGCCTCTCTCAATGAATCCACCACCCAAATCAGATCCTCC**

**33 S S F Q T P Q R T M E E V W K D I N L A S L N E S T T Q I R S S**

**193 TCCTCCTCCTCACCGCTCCTTCACGTTAACCTCCCAAATGGGACCCACCGCCACCGCCCAAACTGCAGAAATATGACCCTTCAAGACTTCCTTGCC**

**65 S S S S P L L H V N L P N G T H R H R P N C R N M T L Q D F L A**

**289 AGACCCTTTGCCCATGACTCACCAGCTGCGGCCACCGCCTTGGTATCCTCCGCTGTCAGCCCTCCCTCTCCTCTTCCTCCATCCTCCCTCAGGCCT**

**97 R P F A H D S P A A A T A L V S S A V S P P S P L P P S S L R P**

**385 CCGAGGTCCGGCTTCGTTGATCATCATCAGCTAAACCCTAATAACCTTTCTAGCAGTGTTTCTAACTCTTTCAGTGGTTGCCCATTTGAGAGCTTG**

**129 P R S G F V D H H Q L N P N N L S S S V S N S F S G C P F E S L**

**481 GCTGCTTCCTCCTCCGGCTTGCCTTCTTTTGGGAATAGATCGTTCACTGAATCTGATCACAGCAATTCCGGAGGAGATGGAAGACATAAGCGGATG**

**161 A A S S S G L P S F G N R S F T E S D H S N S G G D G R H K R M**

**577 ATCAAGAACCGAGAGTCTGCTGCTCGATCTAGGGCAAGAAAGCAGGCTTACACAAATGAGTTGGAGCTAAAAGTTGCACTCTTGATGGAAGAGAAT**

**193 I K N R E S A A R S R A R K Q A Y T N E L E L K V A L L M E E N**

**673 GCAAGGCTCAAAAGACAGCAAGAACATTTAATTGCAGCAGCGTCTCAACAACCCAAAAGGCACAATCTCAATCGATCATCAACAGCTCCATTTTGA**

**225 A R L K R Q Q E H L I A A A S Q Q P K R H N L N R S S T A P F ***

（4）*EjAP1-1-pro*

**1 GACGGCGAAGATGTAAAGCCATACTTATAGTTGGTGGGTATGACATGGCCATAAACGCTCTTTTCAATTTGTAGGGTGGTTGGGGTCCAATTGAAT**

**97 CATTCAATTACAACTCTATTACTACTAATGGATGCCTGGTTGGTGCGTCCTCCCTATTATGCTTTGGTTAGGGAAACAATTTTGAACCCCATATAA**

**193 TTATTCTAGATTTTTTTTTTTTTTTTTTTTTTTTTTTTTTTTTTTTTTTTTTTTTGTGGGTCGAGATAGTCATGCTAGATGATCTACGTAAGAAAA**

**289 TAAGATAAATGTTAAAAGCAAAGTGATGATCAGTTAGAAGAAAGAGACAGCACATCTTAAAATCTGTTTGAGATTACTAAACAAATAATTAACTAA**

**385 AACTTAATTGAGAATCTAAAATTGCTTCTGACATGCCACTCAGTACTACGGTCTGGTGGTATTCCTCTTCACTTGAAAGTGAGAGGTCTTAGGTTC**

**481 GAATCTCGTGGATGATACGTTGCCCATTGTGTGGTTTAGCCGAACTCCCCCTCCACTTAGTGTAAAAATATCGATGTATTAAAAAAAATGCTTCTA**

**577 AAATAGCTAAAAGTCTTTTCTAACAATAAAAAATGTTTTTTTAACTACATTTGAAAGAAAACGTTAATATTTGTAGGTTATAAAAGCATTTTCTAG**

**673 AGAAAGACATATCATTCTTCCCTGTAGCACTAGGATTTTTTTTTTATAATAATATTATACATGTTTTTAATAAAAACACTTCTAACAAAAATAGTC**

**769 TCAACTTCCTTAAACTCTTTATTATTCATATATATATATATATATAACACGATGTTAACATGACATGCATACATATATTTCACTAACAATATGGTA**

**865 ACCAAATGATCCCCTCCTCATTTTATGTAGGAAAAATGAGTTAGCTTAATCATTTTCAGTTCATAAGAGACTAACAGTGTATATCATCAATATATT**

**961 GAATAATATAAACCGTAAAGGAGATAGAGATTAACAATTAAATATTGTTTTCGTGTCAAACTGATCATGAACACATTGTTTTCTTTTTTTTTGGAC**

**1057 AAAGATCATGAACACATTGTTGAAATGTGGGACACCAACGAGCAGTATAATTCCTCTGGCCAAAAACAAACAAATTTTCAACCTACCAATACTAAT**

**1153 GGGGTCAAATCACAGTAATTCTCCCTGTATAAAATGTGGCACTTTGCGTACGTACGTTCGCCGGTTACGAGTCAAGCAATAAGGCAATGGTAACGC**

**1249 TGAAGGCCGTCCGAACAATGCATGCCTGAGACTCTATGAGTCCGAATAGAAGCCAGTAAGTAGTGATCTACACCAGTGTGAAAGAAAAATAAAATT**

**1345 GTGGTTCCAATTACTAGCCCTGTTGGAAAGAGAGAAAACGACTACATAGTGTACCTCAACCAATCAGAAATCGACAACACTTTCTTAGAAATCCCA**

**1441 CCGGTTTTCCCAAAACCACTTGTATTCACAAAACCGCTTTATCTGTCATCAAATCAAATGCATGTATAGATAGCTACTAGCACGTACACTTTCTCA**

**1537 GACACCTTCAGATATTTGGCTAGAAGAAGAAATTTTACATAAATTAAAATTCCCATTAATTTATAACAGTGTTTCTTTCTTTTTGCATATAAATAG**

**1633 CACAGCTTAGTATTTCAGTTTCTGGGTTGTCTTTCTTTTTTTGGTTGTTTGGGTTTTTTGAGAAAGAAAATCAGAGAACAAGCAGTTGAAAGAGTT**

**1729 GAGTGGCAACTGAGAGTCCATAAGTATTTAATC**

（5）*EjAP1-2-pro*

**1 AAAGGGGGGATATAGACCTTACCTCTCTTGAAAAAGAAAATACAAGAAAAATAATGGGTGGACATAGGCCCAACCCCTCTATAAACCTAAAAGGTA**

**97 GGAATTTGCAAGACAAGCTGCAAAACGAAAAGGAAAATCCAAAAAGATTAGGTAAAAAAGAAAATCACACATCAAGAAGGGGAGACAAACTTGTAG**

**193 GCTAGCATACGCAAGAAATCTTAGTGCAGAAGAGAAAAAATCTCCATAGGAGGAGCGGCATGTCAAACAAGTGAAGACGAGATAAGCTATAAATTG**

**289 GCTAATTTGTCAGCAACTGCATTCCCTTCTCGAAATGTATAAGAGCAACAAAATACCATGTTCTGCAATCAGAAAATACAATTTTTCCAACGTGTT**

**385 TGGAGCGACCAAGGAGGAGAGAAAGATCTATAAGCAAAACAAGATATCATACTAGAAGAGTCACTTTCAAGCCATAAATTTTGCCAACCCCGTGCG**

**481 TGGGCTAACTCCACAATAAGGATGATAGCATGAAGCTCCGCATAAAAAGAAGTACGATGCCCCAAACTTAGGGAAAACTACCAAGAAAATAACCCG**

**577 CAGAATCTCGGAAAACCCCAGTAAAAGCCACATAACCCAGGTTACCTTTAGCAAGGTCATCAGTACCAAGAAAATGGTGAAGGATGTCAAAGAACA**

**673 TGGATTATAGATAGAGTTTTACAAGACTCAACCGAGATTACAAGAGAGACTAAAAGTTGCTTACCCAAAATACCCCGTCCGTTACCAGGGTGAAAA**

**769 CTCCAACTTGCCTAATCCATACCGAAATGAATGGCAAAGGGGTGAGAAGGAATAAGGCTTACTTTCAAACTTCACTTTATTACGCATCTTCCAAAT**

**865 AGCCATTAGCAAGAAAAACCATGAAGCTAGCCAAACATTACAAAGTTGTGGAGAAAACCACTTTGACACAAAAGCAAGCGACAAATATGAGAGAGA**

**961 ACCTGTAAGCAGAAAAATAGTTCCAAATTAGGTAGCTAGCCAACACCAAGCCCATTGTGCAAATTCACAACTAAAAAATAAGTGCTCAATAGATTT**

**1057 CGAATTTTTGTAACATAGTTGGCATATTAGAACCAACGGAATGCCCTGTCGCTAAAGTTCATCCTCCGTTGGAAGCTTTTTGAAAGAATCTTTCAA**

**1153 ACTAAAATAGAGTAGCGAGGTGGAATGAAAGGACGCCAAATAATGGAGGCGCAACTCTTAACAGAAAATCAATGACGAACAATTTCATAGCCATCA**

**1249 GATAACAAAATGACGAACAATTTTATTTTTTAAGTTATTAACTTTTTAGCACATATCTCACTATTTGTATAATAGCACGTGATGTACTATTTTGTG**

**1345 TGCTAATCACACTGAAAAATCTCTCATGCCAGAGACTAATGGGGGTCAAATCACGGTAATTTTCCCTAAACAAAAAGTGGCACTGTGCATACGCAC**

**1441 GTCAGCTGGTTACGCGTCGAGAAAAAAAAGTAACGGTAAAGCTGAAGGTCCGTACAATGCATACCTGAGGTTCTTGAGTCCGGCAGGAAGCCGGTA**

**1537 ATAGTGACCTACACCGGTAGGTAAGAAAGATAAAATCGAGGTTTCTTTTTCCTGGCCCCGTTGGAAAGAGAGAAAACGACTACATAGTACACCTCA**

**1633 GCTAATCAGAAGTCGACAACACATCCTTTAAAACCTCACCGGTTTTCCCAAAACCACTTTGATTCACAAAAACCGCTTTATCTATCATCAAATCAA**

**1729 AAGTCTGTAGAGCTAGCTACTAGCACATATACTTTCCCAGGCACCTTCACATATTTGGCTATAAGAAGAAACTTTGCATAAATTAATATTCCCATT**

**1825 AAATTGTAACTATATTTCTTTCTTTTTTGTATATAAATCGACAGCCTACCAGTTCATTTTCTGGGTTGTCTTTCTTTTTCTGGGGGTTTGGGTTGT**

**1921 TTGGTTTTTTTGAGAAAGAAAATCAAAGAACAAGCAGTTGAAAGAGTGAGTGGTAACTGAGAGTCCATTAATATTTAATCA**

（6）*EjTFL1-1-pro*

**1 CTAGCAGCTTGGCAGCAGGCACTCAGAGGGCAGGTGGAGTTCCTTTATGCTCTTGGGCCTCCTGTCTGAGAGAATCTCTGGAAAAATGATGACATT**

**97 CCTGTGTACATTATGCATCCATTCAACACAGTATATTTCCATTTCAAAATTTTTATTACCCTAAATGCCAAAGATGAGAGTTTCCTTTATCAGATT**

**193 GTGAATGCTCAGGAAGAATTCTAGGCATTTTGCTGTTTTTGCTTTATGTTGTTTTATAAAAAAACAGTTATGACTAGGCAAGGAGATATTTTTCAG**

**289 TATGCTCAAAACACAGGTACATACGTTATTATAAAAATAAAGAAGCACTTGAAAAAAGAAACTTCTACCTACTTCTATAATAATATATGACGCAAC**

**385 GTTCCGTATTCCAAATATATTAAAAAATCTCTCATATGCAAGGACCCCAACCATGCAAAATTATATAATGATTTTCTGGCCTATCTATGGAAAAAC**

**481 ATCCCCCTTATTTTTGAAAAAAAAAAATTCAATAATTAGGTTTACAACCAATCAAGAACTAATTCCAAGGGGCCCAATTAATATGTGAGGCACACA**

**577 AATATACAGGATCGGATCACACAAAACATAATTATAATGTGCATTTATCAAAGAAGACTCTTGCTTAGAATCTATGTATAACATTCATTTGGTTAA**

**673 TTTTACAACCAATCAAGAACTAATTCCAAGGGGCCCAATTAATATGTGAGGCACACAAATATACAGGATCGGATCACACAAAACATAATTATAATG**

**769 TGCATTTATCAAAGAAGACTCTTGCTTAGAATCTATGTATAACATTCATTTGGTTAATTTTTAATTGGAGCATAGTATTTACACACCTCTTTTTGC**

**865 CTTTCATACAACATTCTCAATTTTGACCATCCGGCTCGAATGATCATGAATTAATGAAGATCAAATTATTGAAATTAACAAGGAGCTATATGAGAA**

**961 GTAAAGTGAATAATAGCATCCTTTCAAATTACTCTGTGAGTAAAAAAAGTATAAATCTCACATTAATATATAAGATATTGGAGAAAGTTAAGAAAA**

**1057 GTTCCGTTTTTGCTCACCCCTTATAACCCAAAGTAACCGTTACCGCCCTCGTGTCTCAAATTAATTCAACCCAACAACTAGAACAAAAATAAAAAA**

**1153 AGGATTCTTTGCTTTCTTATTAAATTATTGAACTTAAGTTTAAAGATTATTCTTTGTAGAAATTTTGGTTGTTCGGTTTAATATAAAATAATAATC**

**1249 GAACCCAATTTGCTCTCCTTCGTTTATCCTGTAGAAATTTAATATTGATTTGGTTGTTAAGTATTTTGGTTTAGTTTTAAACGGGAACCTGGAATA**

**1345 CAGTGATCTCCTGGATCTTTAGACTCCAAAGGCATTAAGGTTCTATGGTTTGGACCTCCCTATTTCAACCAGTCGTTCGGAAAGATGGTGATTTGC**

**1441 AGTGCCATCGCCTCAGCTTTGTCACGGGCCACGGCCAATTGGGATAAGAAGAGGGAGAGACAGAGATGATGGGAATTGTTTAACGTCAGTATCGAA**

**1537 AATAAATGTTATACTAAATGATGTGGCATATTTCAAGTCATTGGATTTTATATCGGACTAGACTAAGCGAAATACGGAAAAAATTTGGAAAAACTA**

**1633 GACGTAGATATTCGAAAAAAAGCTAAATGTAGAGACTCGTAAAATACAATTAGATTGTAGAGTTGAGCACTTATTTTTTTTAGCTCTATACTCTAA**

**1729 TTTTGCAACGTTGCAACGGCAAGTCTTCTCAGAAAGGGATTTATAAACTTAATTTAATTCAGCCGTATATATAAGAATTTTTCTAGAAGCAAACCA**

**1825 CTAAAAACTCTTGATGTGACGTAGAGAAAGAAGGATGGGAGGTTTGGGACTAACTAGCAATGTCTTTTAGATGCCCCTATTCTAATATTCCTTTAT**

**1921 ACTATTATTCATCTCTTCTCTTAAAACACACTAGATATCTAAAGTAATTAGCTTGCTGTGTGCATGAAAACACTATAAATACAACCATACAAGGAT**

**2017 TCCACTCCCAAGCAATATAAGGAGTGCTATTAGTTCCTCCTGAATTGACTTATCCATTAATCTTTTCTTTTTACACACACACACACACACTCACTC**

**2113 TCTCTCTCTCTCTCTCTCTCTCTCTCTCTCTCTCTCTCTCTCTCTCTCTCTCATAAA**

（7）*EjTFL1-2-pro*

**1 ACGTGCCCACGAGGTGAGGGGGGAGGGTATGGCCAATCAATCTTCCATGCCTAAGTCAATTTCTCTGAAAAGAGAGAATTTTGGAGCTTTTATGTG**

**97 TAGCAAAAGCTTACCAAAAAATGGTGGAGATGGGCTATTTATAGGGAGGCGGCGGAAATGGTAGGGTTTTGCCCTACATGTGGTAGCTTACCATTG**

**193 GCCAGTGTCATCTGTAGGGTAGACAAGGAAAGAATAATCCCAAAGATATTAATTAGAAGATACTTGGGACACTGGCGGAGGCATATTAGGACTAGA**

**289 GTAGTCCTAGGCCTACCGTCACTTGTAAAAATAAGGTTAGTATTACACCTTGCTTTGCAACCCTTCTCAACACGTGAAACAAATCCAATCATTATC**

**385 TATTTTTAATCCACCAAATTCCATCCCAAAATTAAGTATAATATTTCGAATCGTCATTTATCACAAATCAATAACATTCAAATTCAACTCATCATT**

**481 CACTTTTAATTAACAAAAATTGTCTTTCACTTATGCACTGCCAGAGCTATTTCCCTATGCTTCTTCAATTTCATATATTTAACCATTCCAAATCCC**

**577 AAATTTATTGAATAAAGAACAATAAGGTTTGTAAAACTCTATTATCCTCTATAAAACACTTGTTTCTATTCTATCCTACCTCTTACTTTGAGTTTT**

**673 AGGGTTTGCAAATTTTTAGTAATTCTGTCTATTGAGTTTAATAATACTTTCAATAGTTCATGCACAACGGTCTCAAAATATGAAATTCATATTATC**

**769 TATGTATATGTTAATTTTGACTATTTAATTATTTGGATATATATTATGATGTTTTCCATTTGTAATTTTATAATCACTATTCATTTTTTCTTCTTC**

**865 TATACTTGTGTGAGGCCCCTTCTCACAAGAAATCCTAGCTCCGCCACTGACTTGGGATGATAAGGTAAGTATCCTTGATTAATTGTGATGAGGATT**

**961 CCTTACTTGTTTGAGTTGATCTTCAATCAGGAACGTATTAATGATAGGGTTGGATGATTAATCCTTTTTTTAGCAAATTAATCATATTTTAAGGAG**

**1057 TCCAAATCTTCTGCACCCAAAAATCCTTGTGGCCTCTCTGTGCCTTTGTTAATTGGCTTTATTTTTTATTATTTTAATTATTATCAAATTAACATT**

**1153 GATTATATTTGGTTCAATTAACAAAGGCACAGAGATGCCACATGGGTTTTTGGGTGCAGAAGATCTCAATTCTATTTTAAGCCTCTATGGATGTTG**

**1249 AACTGTGCGCGGCAATATTTTGGGGCCTTGCCCATTTAATGAGGGCATTATTGTCTTCTTCGGGAAAAAATTCACATGTCGTCTCCAAGATTTTTA**

**1345 GAATTATTTTTGGCTTCACATATGTTATCACAAAAAATTCTTGCATGCATTATGTGTATAAAATGTGTATAACATTTCTCTGATTAATTTTTAATT**

**1441 ACGTTGTGAGTATAAGCAGTATAAATTATTGCATACAAGCATGTCACTTAATTTTGAGTTTTCTTTTTATTTTTATACACACAACGATATTGTGTG**

**1537 ATGAAGTTTGTCTAGCCCACACAATAGGTTAGCAATAATTTGATATCGAACTCGTCATATAAGAGTTTAATTTAAGACATTTCACTTACAAGTGAA**

**1633 GAAAAATATAATTAGAATGTAGAGTTGACCACTTAATATTCGAGTTAAATACTCTAATTTTTACAACGGTGAGTTCTCTCAAAAAGGGATTTATAT**

**1729 CAGCCATTAAACATATAAGAATGGAAGCAAACCACTAAAAACTCTTGATGTGACATAGAGAAAGAAGGGTGGGAAGTTGGGGAGAGTACCTAGCAA**

**1825 TGTCTTTTAGACGTCCATAAAATCCTTTGTACTATTTTTCTTCTCTTCTTCTCTCAAAACACATTAGCTAGAGATATGAAGTAATTAGCTCGTTGT**

**1921 GTACATGAAAACACTATAAATACACCATACAAGGATTCCACTTCCAAGCAATATAAGAAGTACTACTCTCTTCTCTTAAA**
